# Artesunate induces ferroptosis in gastric cancer by targeting the TFRC-HSPA9 axis for iron homeostasis regulation

**DOI:** 10.1016/j.redox.2025.103867

**Published:** 2025-09-10

**Authors:** Yi Liu, You Yu, Zhihong Luo, Ruoxin Fang, Xiaodong Zhang, Zhengkai Liao, Wenhua Li

**Affiliations:** a Hubei Key Laboratory of Cell Homeostasis, College of Life Sciences, Wuhan University, Wuhan, 430072, PR China; b Wuhan University Shenzhen Research Institute, Shenzhen, 518057, PR China; c Department of Radiation and Medical Oncology, Zhongnan Hospital of Wuhan University, Hubei Key Laboratory of Tumor Biological Behaviors, Hubei Cancer Clinical Study Center, Wuhan, 430071, PR China

**Keywords:** Artesunate, Gastric cancer, Ferroptosis, TFRC, HSPA9

## Abstract

Ferroptosis, a recently characterized form of regulated cell death driven by iron-dependent lipid peroxidation, has emerged as a promising therapeutic strategy for cancer treatment due to its potential for selectively targeting cancer cells. Exploiting FDA-approved drugs to induce ferroptosis offers a novel approach that exploits cancer cells' vulnerabilities in iron metabolism and oxidative stress. Here, we identify artesunate, an antimalarial drug, as a potent inducer of ferroptosis in gastric cancer cells and reveal the transferrin receptor (TFRC) as a key mediator in this process. Notably, our study is the first to demonstrate an interaction between artesunate and TFRC through molecular docking and surface plasmon resonance (SPR) experiments, highlighting a novel mechanism by which artesunate stabilizes TFRC by inhibiting its lysosomal degradation. This stabilization is regulated via the heat shock protein HSPA9, another previously unreported interaction. Disrupting the TFRC-HSPA9 interaction facilitates iron accumulation and lipid peroxidation, hallmark features of ferroptosis, leading to significant cancer cell death. Additionally, in vivo studies confirm artesunate's anti-tumor efficacy, showing marked tumor growth inhibition and minimal systemic toxicity. These findings underscore the therapeutic relevance of targeting ferroptosis in cancer, particularly by leveraging TFRC's role in iron homeostasis. Furthermore, this study expands the understanding of post-translational regulation in ferroptosis, offering a new perspective on the role of artesunate in cancer therapy.

## Graphical abstract

**Figure:**
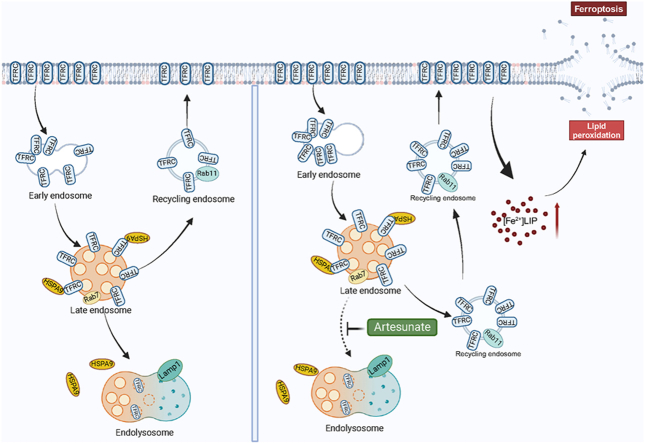

## 1 Introduction

Ferroptosis, a regulated form of cell death first defined in 2012, is driven by iron-dependent lipid peroxidation [1,2]. It is marked by the buildup of reactive oxygen species (ROS) and lipid peroxides, which compromise membrane integrity and lead to cell death [3]. Unlike apoptosis or necrosis, ferroptosis relies on iron and polyunsaturated fatty acids (PUFAs), particularly through both enzymatic and non-enzymatic lipid peroxidation pathways [4]. This process presents a promising avenue for targeting cancer cells, especially those resistant to conventional apoptosis-inducing therapies [5,6]. Cancer cells, known for dysregulated iron metabolism and heightened oxidative stress, are particularly susceptible to ferroptosis [5,7]. For instance, ATM kinase promotes ferroptosis by regulating iron metabolism through NCOA4-mediated ferritinophagy, ensuring iron availability and facilitating lipid peroxide accumulation [8]. Similarly, SOX8 promotes ferroptosis in hepatocellular carcinoma by disrupting iron metabolism and increasing oxidative stress [9]. Therefore, the dysregulation of iron metabolism not only amplifies cancer cell sensitivity to oxidative stress but also creates favorable conditions for ferroptosis.

In response to these insights, researchers are actively exploring methods to induce ferroptosis by targeting iron metabolism and oxidative stress responses to eliminate cancer cells [10]. This investigation has led to the discovery of various inducers capable of triggering ferroptosis, including both experimental compounds and FDA-approved drugs [2,11]. Notably, erastin and RSL3 were among the first identified inducers, selectively triggering ferroptosis in RAS-mutant cells by inhibiting the cystine/glutamate antiporter (system Xc (−)) or directly targeting GPX4 [1,12]. Other inducers, such as sorafenib, sulfasalazine, and artemisinin, promote iron-dependent oxidative damage in cancer cells [6]. For example, ATF2 inhibits sorafenib-induced ferroptosis in gastric cancer by stabilizing SLC7A11 through HSPH1, suggesting that targeting ATF2 could enhance sensitivity to sorafenib [13]. Artemisinin compounds increase intracellular free iron through ferritin degradation and disrupt iron homeostasis, leading to lipid peroxidation [14]. Additionally, the DHODH inhibitor brequinar has been shown to work synergistically with sulfasalazine to induce mitochondrial lipid peroxidation and ferroptosis in GPX4^high^ cancer cells [15]. The clinical potential of ferroptosis lies in its ability to selectively eliminate cancer cells while sparing normal cells [16]. Given that cancer cells often demonstrate a heightened dependence on iron and increased oxidative stress, inducing ferroptosis offers a promising therapeutic avenue with potentially fewer side effects compared to conventional treatments [5,17]. The discovery of small-molecule ferroptosis inducers and the development of combination therapies with existing cancer treatments present exciting opportunities for advancing ferroptosis-based cancer therapies [18].

Artesunate (ART), originally developed as an antimalarial drug from the Chinese herb Artemisia annua, works by generating free radicals through its 1,2,4-trioxane structure that reacts with iron, leading to the destruction of malaria parasites [19,20]. With FDA approval, artesunate is now a first-line treatment for malaria [21]. Its therapeutic potential has since extended to oncology, where it has shown significant anticancer activity in cancers such as liver, breast, and leukemia [[22], [23], [24]]. Artesunate induces several forms of cell death, including apoptosis, autophagy, and ferroptosis [[25], [26], [27]]. It regulates oxidative stress, disrupts cancer signaling pathways, and inhibits angiogenesis, which suppresses cancer cell proliferation [28,29]. When combined with traditional chemotherapy, artesunate enhances treatment efficacy and helps overcome drug resistance [30,31]. Notably, artesunate has emerged as a potential ferroptosis inducer by increasing intracellular iron and triggering lipid peroxidation [26,32,33]. However, the precise mechanisms by which artesunate induces ferroptosis remain unclear and require further investigation.

In this study, we demonstrate for the first time that artesunate induces ferroptosis in gastric cancer cells through its regulation of TFRC, specifically by stabilizing TFRC expression and inhibiting HSPA9-mediated lysosomal degradation of TFRC. Our results show that artesunate disrupts the interaction between TFRC and HSPA9, preventing TFRC degradation and promoting sustained iron influx, which is critical for ferroptosis. Furthermore, in vivo studies reveal significant tumor inhibition with artesunate treatment, with no noticeable toxic side effects in mice. In summary, our study elucidates the mechanism by which artesunate induces ferroptosis through the TFRC-HSPA9 axis, offering a novel approach for cancer therapy.

## 2 Materials and methods

### 2.1 Cell lines and culture

Human gastric cancer cell lines (MGC-803, MKN45, HGC27, AGS, SGC-7901), human gastric mucosal epithelial cells (GES-1), human normal liver cells (L02), and human embryonic lung fibroblasts (MRC-5) were purchased from CCTCC (Wuhan, China). All cells were cultured in DMEM supplemented with 10 % fetal bovine serum (AusGeneX), 1 % streptomycin, and 1 % penicillin, maintained at 37 °C with 5 % CO_2_ in a humidified incubator.

### 2.2 Reagents and antibodies

Artesunate and deferoxamine mesylate (DFO) were obtained from TargetMol (Boston, MA, USA); chloroquine (CQ), 3-methyladenine (3-MA), necrostatin-1 (Nec-1), ferrostatin-1 (Fer-1), N-acetyl-l-cysteine (NAC) and tiron were purchased from Sigma (St. Louis, MO, USA); Z-VAD-FMK was purchased from Selleck (Houston, TX, USA). The following antibodies were purchased: PARP (# 9542, RRID: AB_2160739), caspase 9 (#9502, RRID: AB_2068621), TFRC (#10084-2-AP, CD71), HSPA9 (#A0558, RRID: AB_2757264), ATP1A1 (#14418-1-AP, RRID: AB_2227873), RAB11 A/B (#15903-1-AP, RRID: AB_2173458), LAMP1 (#9091, RRID: AB_2687579), β-Tubulin (#10094-1-AP, RRID: AB_2210695), and LC3B (#L7543, RRID: AB_796155), HA-Tag (#3724, RRID: AB_1549585), DYKDDDDK Tag (#14793, RRID: AB_10950495).

### 2.3 Cell proliferation and viability

Cells were plated at a density of 4 × 10^3^ cells per well in 96-well plates, with each condition replicated in triplicate. After seeding, cells were treated with the indicated pharmacological agents and incubated at 37 °C for 72 h. Total cell counts were conducted to assess proliferation, and cell viability was determined using the trypan blue exclusion assay (ST2780; Beyotime, China). Viability results were expressed as a percentage of viable cells relative to the total cell population.

### 2.4 Western blotting

After a series of treatments, cellular samples were harvested and purified using phosphate-buffered saline (PBS). Cell lysis was performed in a cold environment by adding 1 % sodium dodecyl sulfate (SDS). The lysates were then heated to 95–100 °C for 10 min, followed by centrifugation at 10,000×*g* for 10 min. The supernatant was carefully collected for subsequent analysis. Protein concentrations were quantified using the BCA protein assay kit (23227, Thermo Fisher Scientific, USA). Equal amounts of proteins were separated by SDS-PAGE and transferred onto polyvinylidene fluoride (PVDF) membranes (Millipore). The membranes were then subjected to immunoblotting using specific primary and secondary antibodies. Detection of the bound antibodies was achieved through the application of a chemiluminescent substrate for horseradish peroxidase (HRP) (Millipore).

### 2.5 Apoptosis analysis

Cells were seeded into 6-well plates and treated as described in the corresponding figure legends. For apoptosis analysis, cells were processed according to the manufacturer's protocol using the Annexin V-FITC/PI apoptosis detection kit (#556547, BD Biosciences) and analyzed using a flow cytometer (Beckman).

### 2.6 Colony formation assay

Cell proliferation was assessed using a plate colony formation assay. Briefly, 1000 cells were seeded into each well of a 6-well plate. After allowing the cells to adhere overnight, they were treated with 15 μM artesunate and incubated for 6 days, with regular medium changes. The plates were then gently rinsed and stained with 0.1 % crystal violet (Y268090; Beyotime, China), followed by colony counting.

### 2.7 ROS measurement

To ensure methodological rigor, ROS levels were measured using a combination of fluorescent probes, as advised by Murphy et al. [34]. Cells were stained with 1 μM DCFH-DA (HY-D0940; MedChemExpress, USA) or BODIPY™ 581/591 C11 (D3861; Thermofisher, USA) in serum-free medium at 37 °C for 30 min to measure general ROS levels. Following staining, the cells were washed twice with PBS and analyzed by flow cytometry to quantify ROS levels. Additionally, superoxide anion levels were detected by incubating the cells with 5 μM dihydroethidium (DHE) probe (S0064S; Beyotime, China) for 30 min at 37 °C. The DHE probe specifically reacts with superoxide anions to produce a fluorescent product (2-hydroxyethidium), which can be detected at 535/610 nm. To assess mitochondrial superoxide, cells were treated with 5 μM MitoSOX Red (HY-D1055; MedChemExpress, USA) for 30 min at room temperature in the dark. This probe selectively accumulates in the mitochondria and emits fluorescence at 510/580 nm in the presence of superoxide. After staining with DHE and MitoSOX Red, cells were washed twice with PBS, and fluorescence intensity was analyzed by flow cytometry to quantify the levels of superoxide in the cytoplasm and mitochondria, respectively.

### 2.8 Measurement of Fe^2+^ levels

The concentration oironf Fe^2+^ was measured using an iron assay kit (A039-2-1; Nanjing Jiancheng Bio, China), following the manufacturer's instructions. Briefly, cells were lysed with RIPA buffer for 30 min and centrifuged at 12,000×*g* for 10 min at 4 °C. The resulting supernatant was analyzed according to the kit protocol, with absorbance measured at 520 nm using a microplate reader.

### 2.9 Malondialdehyde (MDA) assay

The cellular or tissue specimens were processed according to the provided guidelines. The concentrations of MDA (S0131S; Beyotime, China) were then quantified using the respective assay kits, following the manufacturer's protocols.

### 2.10 GSH-Px activity assay

The activity of glutathione peroxidase (GSH-Px) was measured using the Total Glutathione Peroxidase Assay Kit (S0058; Beyotime, China), following the manufacturer's instructions.

### 2.11 Quantitative RT‒PCR

Total RNA was extracted from cell lysates using the Total RNA Kit I (R6834-02; Omega Bio-Tek, USA). The extracted RNA was then reverse transcribed into cDNA using a cDNA synthesis kit (5081955001; Roche Applied Science; Swiss). Quantitative real-time PCR (RT-PCR) was performed on the 7500 Fast Real-Time PCR System, with amplification signals monitored by the increased fluorescence of SYBR Green (KK4621; Roche Applied Science, Swiss). β-Tubulin served as the internal control for normalization, and relative gene expression levels were calculated using the comparative CT (ΔΔCT) method. Primer sequences are provided in Table S1.

### 2.12 Transmission electron microscopy

Cells were cultured in 10 cm dishes until reaching 80 % confluence and fixed with electron microscopy fixative for 1 h. After washing three times, the cells were scraped into an EP tube and centrifuged at 4 °C to form a compact pellet. The pellet was then stained with osmium tetroxide on ice in the dark for 1 h, washed with uranyl acetate, and stained overnight at room temperature in the dark. Following washing with ddH_2_O and soaking in 50 % ethanol, the cells underwent gradient dehydration. Cells were incubated in propylene oxide and resin mixtures, first in a 1:1 mixture for 4 h, followed by a 1:2 mixture overnight, both protected from light. Finally, cells were placed in 100 % resin for 4 h, embedded, and cured at 65 °C for 48 h before sectioning for electron microscope observation.

### 2.13 Plasmids and Lentiviral transfection

To achieve stable gene silencing, single-stranded oligonucleotides and their complementary strands were synthesized, with the sequences detailed in Supplementary Table S1. The sense and antisense strands were annealed and cloned into pLKO.1 plasmids. For expression vector construction, cDNA encoding an HA-tagged HSPA9 or a 3 × Flag-tagged TFRC was inserted into the pcDNA3.1 vector. To construct vectors for fluorescent protein expression, TFRC cDNA was inserted into the pEGFP-N1 vector (#172281, Addgene), while HSPA9 cDNA was cloned into the pSicoR-mCherry vector (#21907, Addgene). Both full-length and truncated versions of human TFRC were cloned into Flag-tagged pcDNA3.1. All plasmids were validated by bidirectional sequencing to confirm the presence of the desired mutation and the absence of unintended mutations.

HEK-293T cells were co-transfected with either control or target plasmids (10 μg each), along with the packaging plasmid psPAX2 (5 μg) and envelope plasmid pMD2.G (5 μg), for 48 h. The resulting viral supernatants were used to infect the cells, followed by selection in the presence of puromycin (2 μg/mL; Sigma-Aldrich, USA).

### 2.14 Immunoprecipitation

Following transfection, cells were cultured in 10 cm dishes for 36 h and lysed using 800 μL of RIPA buffer (P0013C; Beyotime, China) supplemented with a protease and phosphatase inhibitor cocktail. The dishes were cooled on ice and incubated with lysis buffer for 10 min. Cell lysates were scraped, collected, and centrifuged at 10,000×*g* for 10 min at 4 °C. To digest genomic DNA, DNase (#79254, QIAGEN) was added to the lysates and incubated at room temperature for 10 min. A 60 μL aliquot of the lysate was mixed with 20 μL of 4 × SDS buffer and heated at 98 °C for 10 min for protein quantification. The samples were then stored at −80 °C for subsequent Western blot analysis.

For immunoprecipitation, 1 μg of the primary antibody was added to the lysate and incubated overnight with rotation (20 s per rotation) at 4 °C. In parallel, 20 μL of rProtein A/G Beads 4FF (SA032025, Smart-Lifesciences) were blocked with 1 % BSA in 200 μL of TBS, under the same conditions. After overnight blocking, the beads were washed and combined with the antibody-lysate mixture, rotating for 3 h at 4 °C. The supernatant was discarded, and the beads were washed six times with 1 mL of wash buffer (50 mM Tris-HCl, pH 7.5, 150 mM NaCl, 5 % glycerol, 0.1 % Triton X-100), rotating for 5 min at 4 °C each time. The beads were then boiled in 60 μL of SDS buffer at 95 °C for 10 min for Western blot analysis. Both input and immunoprecipitated samples were subjected to western blotting for further analysis.

### 2.15 Mass spectrometry analysis

Immunoprecipitation was performed as previously described. Briefly, MKN45 cells transfected with the target plasmid were lysed, and the lysates were incubated with rProtein A/G Beads 4FF for antibody binding. The immunoprecipitated samples were washed, eluted, and subjected to SDS-PAGE under reducing conditions for subsequent mass spectrometry analysis.

Following immunoprecipitation, the eluted samples were reconstituted in 0.1 % formic acid in water/acetonitrile and subjected to proteolytic digestion. The resulting peptides were purified using C18 solid-phase extraction columns and reconstituted in an MS-compatible solvent. LC-MS/MS analysis was performed using a Thermo Scientific Q Exactive Plus mass spectrometer (Thermo Fisher Scientific). Raw data were processed and analyzed using Proteome Discoverer v.1.3 (Thermo Fisher Scientific) to identify and quantify peptides, followed by bioinformatic analysis.

### 2.16 Immunofluorescence

Cell slides were placed in a 12-well plate, and 50,000 cell per well were seeded to allow for adherence. After 48 h of treatment with DMSO or artesunate, or transfection with the EGFP-TFRC plasmid, cells were fixed with 4 % paraformaldehyde for 20 min, followed by three washes with PBS (4 min per wash). Cells were then permeabilized with 1 % Triton X-100 and blocked with 5 % BSA in PBS for 20 min. Primary antibodies were diluted 1:100 in the blocking solution and incubated with the cells at 4 °C for 16 h. Following incubation, cells were washed three times with PBS (4 min each). Secondary antibodies, diluted 1:100 in PBS, were added and incubated for 1 h at 37 °C, followed by three additional PBS washes. After staining with DAPI (HY-D0814; MedChemExpress, USA) at a 1:1000 dilution for 5 min and three more PBS washes, the slides were inverted onto sterile microscope slides. The samples were then analyzed using a confocal microscope, and colocalization was assessed using the colocalization tool in ImageJ.

### 2.17 Immunofluorescence for TFRC–lysosome colocalization

To investigate the subcellular localization of TFRC relative to lysosomes, cells were cultured on coverslips and transfected with the EGFP-TFRC plasmid for 24 h. Following transfection, cells were incubated with the lysosome-specific live-cell dye LysoTracker® Deep Red (L12492, Invitrogen, USA) according to the manufacturer's instructions. After dye incubation, cells were washed thoroughly with phosphate-buffered saline (PBS) to remove excess dye. Subsequently, cells were stained with DAPI to visualize the nuclei. After washing, cells were directly imaged without fixation using a confocal microscope to observe the dynamic localization of TFRC and its colocalization with lysosomes. The expression of EGFP-TFRC and its distribution in relation to lysosomes were assessed. Colocalization analysis of TFRC and lysosomes was quantitatively performed using the Coloc2 plugin in ImageJ, based on Pearson's correlation coefficient.

### 2.18 Subcellular fractionation for lysosomes, endosomes, and plasma membrane proteins

Protein extraction for lysosomes, endosomes, and plasma membrane was performed using the Subcellular Protein Fractionation Kit (BestBio, China; Cat. Nos. 31452, 36712, 3116) according to the manufacturer's instructions. Briefly, cells were harvested and washed with cold PBS. The cell pellets were resuspended in the provided extraction buffers, homogenized, and subjected to sequential centrifugation steps at different speeds to separate the lysosomal, endosomal, and plasma membrane fractions. The resulting protein fractions were collected, quantified, and stored at −80 °C for subsequent Western blot analysis.

### 2.19 Molecular docking analysis

To explore the potential interaction between artesunate and TFRC as well as HSPA9, molecular docking analysis was performed using AutoDock Vina (version 1.1.2). The three-dimensional (3D) structure of the TFRC protein was obtained from the Protein Data Bank (https://www.rcsb.org/). Artesunate was modeled as a small molecule, and its structure was downloaded from the PubChem database (https://pubchem.ncbi.nlm.nih.gov/). Prior to docking, the receptor protein structure was processed using PyMOL (version 2.5.4) to remove water molecules, bound ions, and any small molecules, ensuring that only the protein structure was used in the docking analysis.

The docking grid box was defined using AutoDockTools (version 1.5.7) based on the predicted binding site of TFRC. The grid center coordinates (X, Y, Z) and box dimensions were carefully adjusted to fully encompass the active site while minimizing unnecessary space, thereby optimizing docking efficiency. A **config.txt** file was generated containing these parameters along with other settings required by AutoDock Vina. Docking simulations were executed by running the following command in the terminal:

bash

vina --config **config.txt**.

This command was used to generate nine docking models, from which the conformation with the lowest binding energy (most negative value) was selected as the best model for further analysis. The best docking mode exhibited a binding affinity of −7.2 kcal/mol, indicating a strong interaction between artesunate and TFRC. Visualization and analysis of the docking results were conducted using PyMOL. The best binding conformation revealed three hydrogen bonds between artesunate and TFRC, further supporting the stability of the predicted complex (Fig. 4I).

### 2.20 Surface plasmon resonance (SPR) assay

SPR analysis was performed using the Biacore 8K system (Cytiva, Marlborough, MA, USA). Recombinant human TFRC protein was immobilized on a Series S CM5 sensor chip (GE Healthcare Life, Chicago, USA) through standard amine coupling according to the manufacturer's instructions. HBS-EP^+^ buffer (10 mM HEPES, 150 mM NaCl, 3 mM EDTA, 0.05 % v/v P20, pH 7.4) was used as the running buffer, and all experiments were conducted at 25 °C.

Artesunate was prepared at varying concentrations (0.3125, 0.625, 1.25, 2.5, 5, and 10 μM) in running buffer and injected into the system. The flow rate was set to 30 μL/min. The association phase lasted for 60 s, followed by a dissociation phase of 90 s. Sensorgrams recorded during the experiment were analyzed to obtain the binding kinetics. Interaction parameters, including the association rate constant (Ka), dissociation rate constant (Kd), and equilibrium dissociation constant (KD), were calculated using Biacore Evaluation Software (Version 2.0).

### 2.21 In vivo studies

The in vivo experiments were approved by the Wuhan University Committee on Ethics in the Care and Use of Laboratory Animals and in accordance with the Guidelines for the Care and Use of Laboratory Animals published by the US National Institutes of Health. Female nude nu/nu BALB/c mice, aged 4–5 weeks, were obtained from Hunan SJA Laboratory Animal Co., Ltd. (Changsha, China). The animals were housed in a controlled-temperature environment with 12-h light/dark cycles, high-quality dry wood chip bedding, and 4–5 mice per cage. They had continuous access to food and water. Prior to euthanasia by cervical dislocation, the mice were anesthetized with isoflurane, and all efforts were made to minimize the number of animals used and reduce distress during the experiments. The mice were randomly assigned to different experimental groups.

Xenograft tumors were induced using a method described by Liu et al. [23]. MKN45 shNC and MKN45 shTFRC cells (6 × 10^6 each) were injected subcutaneously into the right flank of each mouse under isoflurane anesthesia. Mice were kept warm on a thermostatically controlled blanket (37.0 °C ± 0.5 °C) with continuous monitoring of breathing. Tumor growth was monitored daily, and the mice were assigned to four treatment groups. Artesunate was prepared in PBS containing 10 % DMSO, 45 % polyethylene glycol, 5 % Tween 80, and saline, and administered intraperitoneally. Mice with xenograft tumors received either vehicle control or 100 mg/kg artesunate daily. Tumor volumes were calculated using the formula: volume (cm^3) = π/6 × larger diameter × (smaller diameter) ^2. At the end of the treatment period, blood samples were collected for toxicological studies, and the mice were humanely euthanized by cervical dislocation following isoflurane anesthesia. Selected xenograft tumors were used for further analyses: five for protein assays and five for histopathological examinations, performed under blinded conditions.

### 2.22 Statistical analyses

Unless otherwise stated, all experiments were conducted at least three times under blinded and randomized conditions. Data were analyzed using GraphPad Prism version 8.0.2 (GraphPad Software Inc.) and Microsoft Excel 2019 (Microsoft Corp.). Statistical significance between two groups was assessed using an unpaired *t*-test, while comparisons involving more than two groups were evaluated using one-way or two-way ANOVA followed by multiple comparison tests against control groups. A *P* value < 0.05 was considered statistically significant. To minimize variability, data were normalized, with control values set as 100 % or used as reference units for comparison.

## 3 Results

### 3.1 Artesunate primarily induced ferroptosis in gastric cancer cells

**Fig. 1:**
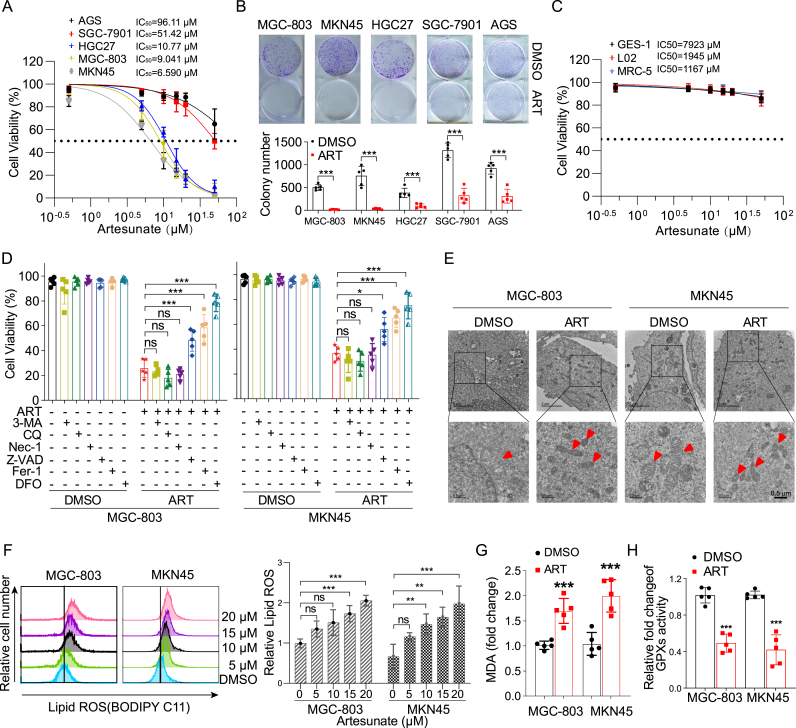
Artesunate exerts anti-gastric cancer effects by inducing ferroptosis (A) The effect of artesunate (0–50 μM) on cell viability. Gastric cancer cell lines (AGS, SGC-7901, HGC27, MGC-803, and MKN45) were treated with the indicated concentrations of artesunate for 72 h, and cell viability was then analyzed by trypan blue dye exclusion assays. Data are presented as the means ± SD, n = 3 independent experiments. (B) Colony formation assay of MGC-803, MKN45, HGC27, SGC-7901 and AGS cells treated with 15 μM artesunate for 6 days. After staining with crystal violet, the number of colonies was quantified. Data are presented as the means ± SD, n = 5 independent experiments. (C) Cell viability of GES-1, L02 and MRC5 cells after 72 h of 15 μM artesunate treatment. Data are presented as the means ± SD, n = 5 independent experiments. (D) Cells pre-treated with 1.5 mM 3-Methyladenine (3-MA), 10 μM Chloroquine (CQ), 50 μM Necrostatin-1 (Nec-1), 100 μM Z-VAD-FMK, 10 μM ferrostatin-1 (Fer-1) or 10 μM DFO for 3 h, followed by the addition of 15 μM artesunate for a 72-h treatment, are subjected to cell viability assays. Data are presented as the means ± SD, n = 5 independent experiments. (E) Cell morphology was examined using transmission electron microscopy following treatment with artesunate (15 μM) for 48 h. (F) MGC-803 and MKN45 cell lines treated with varying concentrations of artesunate for 24 h undergo flow cytometry to detect changes in intracellular lipid ROS. Data are presented as the means ± SD, n = 3 independent experiments. (G) The MGC-803 and MKN45 cell lines, subjected to 15 μM artesunate for 24 h, are then collected, lysed with RIPA buffer, and intracellular malondialdehyde (MDA) levels are measured using a kit. Data are presented as the means ± SD, n = 5 independent experiments. (H) Treatment of MGC-803 and MKN45 cell lines with 15 μM artesunate for 48 h, followed by cell collection, lysis with RIPA buffer, and extraction of the protein supernatant, facilitates the measurement of changes in total glutathione peroxidases (GPXs) activity within the cells using a kit. Data are presented as the means ± SD, n = 5 independent experiments. Significance was determined using one-way ANOVA or two-way ANOVA (*∗P* < 0.05, ∗*∗P* < 0.01, ∗∗*∗P* < 0.001).

Accumulating evidence has demonstrated that artesunate holds significant potential for the prevention and treatment of gastric cancer in clinical settings, effectively inhibiting the proliferation and viability of gastric cancer cells [35,36]. To evaluate the anti-cancer effects of artesunate, we tested its impact on the viability of five gastric cancer cell lines, including MGC-803, MKN45, HGC27, SGC-7901, and AGS. Consistent with these findings, our study revealed that artesunate exhibited potent cytotoxic effects against these cell lines, with IC_50_ values of 6.59 μM, 9.041 μM, 10.77 μM, 51.42 μM, and 96.11 μM, respectively (Fig. 1A and B). In contrast, artesunate exhibited minimal cytotoxicity in non-tumorigenic cell lines, including GES-1, L02, and MRC-5, with IC_50_ values exceeding 1000 μM (Fig. 1C and Fig. S1A). These results suggest that artesunate preferentially inhibits the growth of gastric cancer cells compared to non-tumorigenic cell lines.

To investigate the mechanisms underlying artesunate's selective cytotoxicity, we examined several well-established forms of regulated cell death using the two most sensitive gastric cancer cell lines, MGC-803 and MKN45, as experimental models. Various cell death inhibitors were employed, including autophagy inhibitors (3-Methyladenine [3-MA] and chloroquine [CQ]), an apoptosis inhibitor (Z-VAD-FMK), a necroptosis inhibitor (Nec-1), and a ferroptosis inhibitor (Fer-1). Given that our prior findings demonstrated artesunate's ability to regulate iron homeostasis in cells [23], we also included the iron chelator deferoxamine (DFO) to explore the potential role of ferroptosis. Earlier studies have shown that artesunate can induce apoptosis in gastric cancer cells [36]; however, our findings indicate that ferroptosis is the predominant pathway. In our study, Z-VAD-FMK only modestly improved cell viability compared to DMSO (Fig. 1D), suggesting a limited involvement of apoptosis. Flow cytometry analysis confirmed low proportions of early and late apoptotic cells, and Western blot analysis of PARP expression revealed only a slight increase in PARP cleavage bands, even with high concentrations of artesunate (Fig. S1B and C). In contrast, the rescue effects of DFO and Fer-1 were considerably more pronounced than those of Z-VAD-FMK (Fig. 1D), underscoring ferroptosis as a possible primary mechanism underlying artesunate's cytotoxic effects in gastric cancer cells.

Building on these findings, we further investigated key ferroptotic markers to confirm that artesunate predominantly induces ferroptosis in gastric cancer cells. Transmission electron microscopy (TEM) analysis revealed typical ferroptotic features in MGC-803 and MKN45 cells treated with ART, including condensed mitochondrial membranes and reduced mitochondrial size, which were absent in DMSO-treated controls (Fig. 1E). Consistent with these morphological changes, lipid peroxidation levels, a hallmark of ferroptosis, were significantly elevated in ART-treated cells, as evidenced by increased BODIPY C11 staining in both cell lines across various ART concentrations (Fig. 1F). In contrast, no significant change in lipid ROS levels was observed in GES-1 cells treated with ART (Fig. S1D). Furthermore, measurement of malondialdehyde (MDA) levels demonstrated a substantial rise in MDA content in ART-treated cells compared to controls, indicating oxidative damage associated with lipid peroxidation (Fig. 1G). Previous studies show dihydroartemisinin induces ferroptosis by increasing intracellular free iron and inhibiting GPX4 activity [14]. We evaluated GPX4 activity in our system: ART treatment significantly reduced GPX4 activity in MGC-803 and MKN45 cells compared to DMSO controls (Fig. 1H). Collectively, these findings corroborate that ART predominantly induces ferroptosis in gastric cancer cells.

### 3.2 ROS and iron homeostasis were involved in artesunate-induced cell ferroptosis

**Fig. 2:**
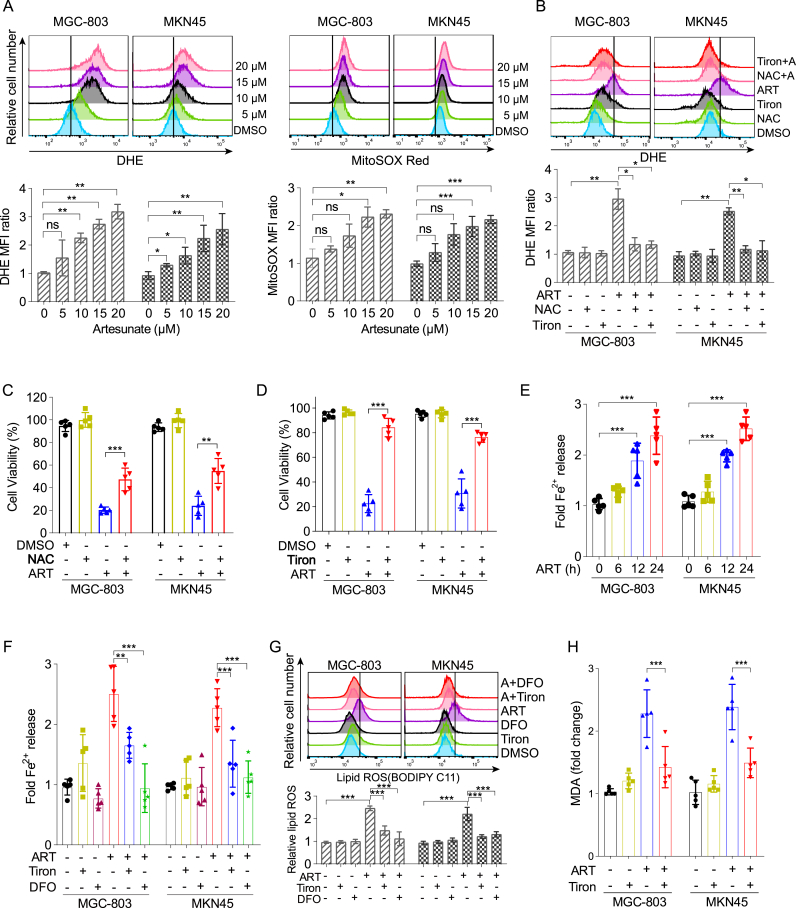
The Role of ROS and iron homeostasis in the induction of ferroptosis by artesunate (A) MGC-803 and MKN45 cells were treated with different concentrations of artesunate (0–20 μM) for 24 h. The levels of intracellular superoxide (detected by DHE) and mitochondrial superoxide (detected by MitoSOX Red) were assessed by flow cytometry. Representative flow cytometric histograms (upper panel) and quantitative analysis of the MFI ratio (lower panel) are shown. Data are presented as the means ± SD, n = 3 independent experiments. (B) Cells were treated with the indicated conditions for 24 h: DMSO, 15 μM ART, 1 mM Tiron, 5 mM NAC, ART + NAC, and ART + tiron. ROS levels were measured by DHE fluorescence intensity. (Top) Representative histograms showing DHE fluorescence in MGC-803 and MKN45 cells. (Bottom) Quantification of mean fluorescence intensity (MFI) ratio from flow cytometry analysis. Data are presented as the means ± SD, n = 3 independent experiments. (C) Cell viability in MGC-803 and MKN45 cells pre-treated with 5 mM NAC for 3 h, then treated with 15 μM artesunate for 72 h. Data are presented as the means ± SD, n = 5 independent experiments. (D) Cell viability following 3-h pre-treatment with 1 mM tiron and 72-h treatment with 15 μM artesunate in MGC-803 and MKN45 cells. Data are presented as the means ± SD, n = 5 independent experiments. (E) Intracellular ferrous ion levels in MGC-803/MKN45 cells treated with 15 μM artesunate for varying durations, measured using a tissue iron detection kit. Data are presented as the means ± SD, n = 5 independent experiments. (F) Intracellular ferrous ion levels post 3-h pre-treatment with 1 mM tiron or 10 μM DFO, followed by 24-h treatment with artesunate, assessed using a tissue iron detection kit. Data are presented as the means ± SD, n = 5 independent experiments. (G) Changes in intracellular lipid ROS in cells pre-treated with 1 mM tiron or 10 μM DFO for 3 h, followed by 24-h treatment with 15 μM artesunate, measured using flow cytometry. A: artesunate. Data are presented as the means ± SD, n = 3 independent experiments. (H) Alterations in MDA content in cells pre-treated with 1 mM tiron for 3 h, then treated with 15 μM artesunate for 24 h. Data are presented as the means ± SD, n = 3 independent experiments. Significance was determined using one-way ANOVA or two-way ANOVA (*∗P* < 0.05, ∗*∗P* < 0.01, ∗∗*∗P* < 0.001).

ROS play a critical role in various cell death pathways, including apoptosis, autophagy, and ferroptosis, and their involvement in the therapeutic efficacy of artesunate has been extensively documented in both malaria and cancer contexts [32,37,38]. To further evaluate ART-induced oxidative stress, we measured total ROS (DCFH-DA), mitochondrial ROS (MitoSOX Red), and superoxide levels (DHE) in gastric cancer cells (MGC-803 and MKN45) and gastric epithelial cells (GES-1). ART treatment markedly increased all three ROS indicators in a dose-dependent manner in gastric cancer cells (Fig. 2A and Fig. S2A). Interestingly, a slight increase in both mitochondrial ROS (MitoSOX Red) and superoxide (DHE) levels was also observed in GES-1 cells, particularly at the 20 μM concentration (Fig. S2B). These results suggest that while ART induces oxidative stress in both cancer and non-cancer cells, the response appears to be more pronounced in the cancerous cell line. This ROS accumulation was effectively neutralized by the antioxidants N-acetyl-l-cysteine (NAC) and tiron, a cell-permeable superoxide scavenger and iron chelator commonly used to reduce oxidative stress (Fig. 2B and Fig. S2C and D). Consistently, the cytotoxicity of artesunate was attenuated by both NAC and tiron, further supporting the involvement of ROS in artesunate's mechanism of action (Fig. 2C and D). Building on our prior research indicating that artesunate modulates iron homeostasis to promote differentiation in leukemia cells [23], we further explored the role of iron in gastric cancer by measuring the intracellular labile iron pool. As shown in Fig. 2E, artesunate treatment resulted in a time-dependent increase in Fe^2+^ levels, which was significantly reduced by tiron and DFO (Fig. 2F). These iron chelators also effectively suppressed lipid peroxidation, as evidenced by reduced lipid ROS and MDA accumulation (Fig. 2G and H). Thus, these results suggest that artesunate promotes ferroptosis in gastric cancer cells by modulating iron homeostasis and driving ROS accumulation, thereby highlighting ROS as a pivotal factor in artesunate's anti-cancer efficacy.

### 3.3 TFRC as a key mediator in artesunate-induced cell ferroptosis

**Fig. 3:**
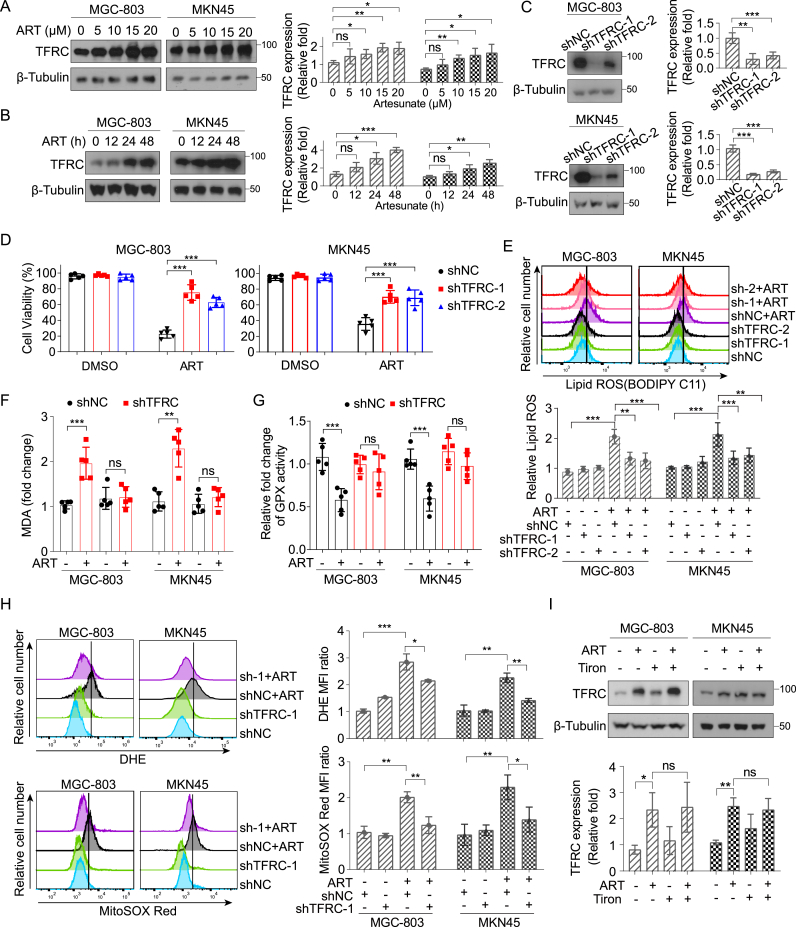
TFRC Acts as a Central Mediator in Artesunate-Induced Ferroptosis (A) Expression of TFRC in MGC-803 and MKN45 cells treated with 0–20 μM artesunate for 48 h, analyzed by Western blot. Data are presented as the means ± SD, n = 3 independent experiments. (B) TFRC expression in MGC-803 and MKN45 cells treated with 15 μM artesunate for 0–48 h, assessed by Western blot. Data are presented as the means ± SD, n = 3 independent experiments. (C) Construction of TFRC knockdown vectors, transfected into high TFRC-expressing MGC-803 and MKN45 cell lines, with knockdown efficiency verified by Western blot. Data are presented as the means ± SD, n = 3 independent experiments. (D) Cell viability in the knockdown cell lines treated with 15 μM artesunate for 72 h, evaluated using trypan blue staining. Data are presented as the means ± SD, n = 5 independent experiments. (E) Flow cytometry analysis of changes in lipid reactive oxygen clusters in stable TFRC knockdown MGC-803 and MKN45 cell lines treated with 15 μM artesunate for 24 h. Data are presented as the means ± SD, n = 3 independent experiments. (F) MDA content in stable TFRC knockdown cell lines treated with 15 μM artesunate for 24 h. Data are presented as the means ± SD, n = 5 independent experiments. (G) Activity changes of total glutathione peroxidases (GPXs) in stable TFRC knockdown MGC-803 and MKN45 cell lines treated with 15 μM artesunate for 48 h, measured using a kit. Data are presented as the means ± SD, n = 5 independent experiments. (H) Changes in intracellular ROS levels in stable TFRC knockdown cell lines treated with 15 μM artesunate for 24 h, evaluated by flow cytometry using DHE and mitoSOX staining. Data are presented as the means ± SD, n = 3 independent experiments. (I) Changes in TFRC expression in cell lines pre-treated with ROS scavenger tiron for 3 h, followed by 15 μM artesunate treatment for 48 h. Data are presented as the means ± SD, n = 3 independent experiments. Significance was determined using one-way ANOVA or two-way ANOVA (*∗P* < 0.05, ∗*∗P* < 0.01, ∗∗*∗P* < 0.001).

Iron homeostasis is essential for maintaining organism health and functionality [39]. Within this regulatory system, the transferrin receptor is crucial for facilitating the uptake of iron ions into cells, which is vital for processes like cell proliferation, metabolism, apoptosis, and ferroptosis [40,41]. To explore whether there is a correlation between TFRC expression and sensitivity to artesunate in gastric cancer, we conducted an initial analysis of TFRC expression across five gastric cancer cell lines. We observed a correlation between TFRC expression levels and the responsiveness of these cells to artesunate, with higher TFRC expression associated with increased sensitivity (Fig. S3A). Artesunate treatment further induced a dose- and time-dependent upregulation of TFRC in sensitive cell lines, particularly in MGC-803 and MKN45 (Fig. 3A and B).

**Fig. 4:**
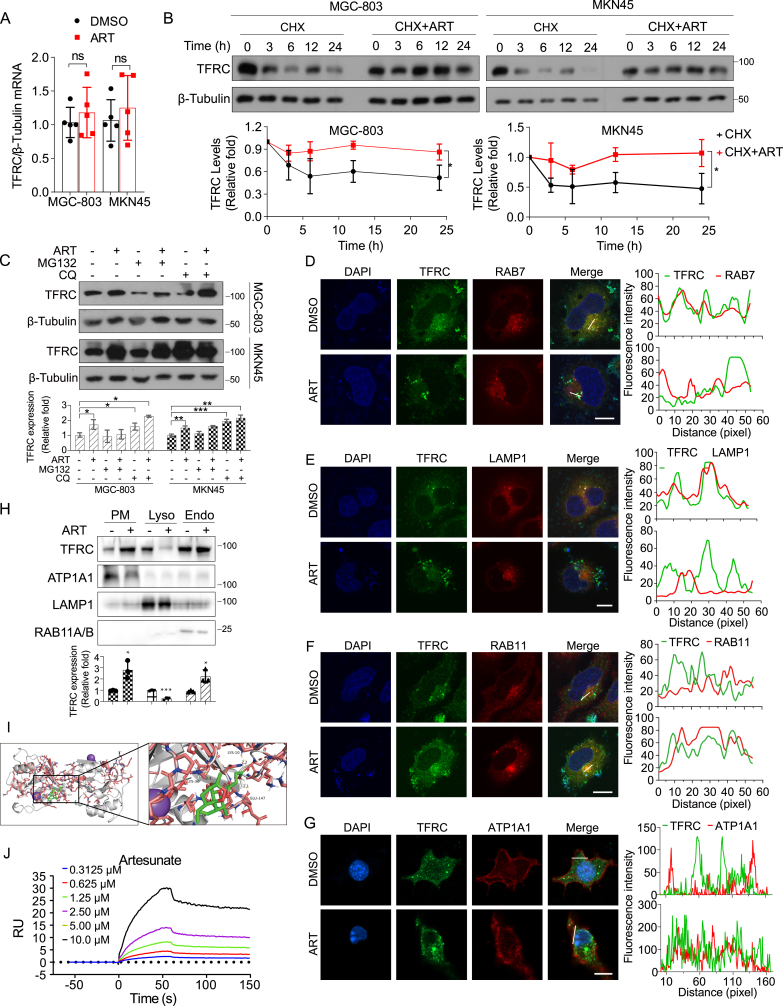
Artesunate inhibits the degradation of TFRC via the endosome-lysosome pathway. (A) Relative TFRC mRNA expression levels in MGC-803 and MKN45 cells after treatment with 15 μM artesunate for 24 h were determined by quantitative PCR. Data are presented as the means ± SD, n = 5 independent experiments. (B) Half-life analysis of TFRC in MGC-803 and MKN45 cells treated with cycloheximide (2 mM) or cycloheximide plus artesunate for varying durations (0–24 h). Data are presented as the means ± SD, n = 3 independent experiments. (C) Western blot analysis of TFRC protein levels in MGC-803 and MKN45 cells after pretreatment with the proteasome inhibitor MG132 (5 μM) or lysosome inhibitor CQ (10 μM). Data are presented as the means ± SD, n = 3 independent experiments. (D) Colocalization of TFRC with RAB7 observed by immunofluorescence staining in cells treated with 15 μM artesunate for 48 h. Scale bar = 5 μm. (E) Colocalization of TFRC with LAMP1 observed by immunofluorescence staining in cells treated with 15 μM artesunate for 48 h. Scale bar = 5 μm. (F) Colocalization of TFRC with RAB11 observed by immunofluorescence staining in cells treated with 15 μM artesunate for 48 h. Scale bar = 5 μm. (G) Colocalization of TFRC with ATP1A1 observed by immunofluorescence staining in cells treated with 15 μM artesunate for 48 h. Scale bar = 5 μm. (H) Subcellular fractionation analysis was performed to assess the impact of 15 μM artesunate treatment for 24 h on TFRC localization. PM: Plasma Membrane; Lyso: Lysosome; Endo: Endosome. (I) AutoDock was used to generate nine binding models. PyMOL was then employed to visualize the best binding mode, identifying three hydrogen bonds between artesunate and residues Lys-10, Lys-36, and Glu-147. (J) Surface plasmon resonance (SPR) analysis of the binding between TFRC and artesunate. Binding kinetics were assessed at various concentrations of artesunate. Significance was determined using one-way ANOVA or two-way ANOVA (*∗P* < 0.05, ∗*∗P* < 0.01, ∗∗*∗P* < 0.001).

To delineate TFRC's role in artesunate-induced cytotoxicity, we performed stable knockdown and overexpression of TFRC in these cells, followed by artesunate treatment. Knockdown of TFRC significantly attenuated the reduction in cell viability induced by artesunate (Fig. 3C and D). Moreover, TFRC knockdown reduced intracellular ferrous iron levels, lipid ROS accumulation, and MDA content, while partially restoring GPX activity (Fig. 3E–G and Fig. S3B), indicating suppression of artesunate-induced ferroptosis. Conversely, TFRC overexpression in artesunate-resistant AGS and SGC-7901 cells was confirmed by western blotting (Fig. S3C). Overexpression of TFRC enhanced artesunate-induced cell death and increased intracellular Fe^2+^ levels as well as lipid ROS accumulation (Fig. S3C–E), further supporting a pro-ferroptotic role of TFRC under artesunate treatment.

To further clarify the relationship between TFRC and ROS production, we measured intracellular ROS levels in TFRC knockdown cells treated with artesunate. Results indicated a significant decrease in ROS production with TFRC knockdown, whereas ROS scavenging did not alter TFRC expression, suggesting that ROS generation precedes TFRC upregulation in our treatment model (Fig. 3H, I and Fig. S3F). These findings support the hypothesis that artesunate induces ferroptosis in gastric cancer cells by modulating TFRC, thereby disrupting iron homeostasis and driving ROS accumulation.

### 3.4 Artesunate inhibited lysosome-dependent degradation of TFRC

TFRC is a constitutively cycling receptor, undergoing clathrin-mediated endocytosis regardless of ligand binding. Its expression is tightly regulated at the protein level through mRNA stability, which is modulated by intracellular iron levels. To investigate the impact of ART on TFRC, we first assessed TFRC mRNA levels in MGC-803 and MKN45 gastric cancer cells treated with artesunate. The results showed no significant change in TFRC mRNA levels (Fig. 4A), indicating that ART does not affect TFRC at the transcriptional level. Next, we examined the effect of ART on TFRC protein stability through a time-point analysis using Western blot in the presence of cycloheximide (CHX), a protein synthesis inhibitor. The analysis revealed that ART prolonged the half-life of TFRC, suggesting that ART affects TFRC at the protein level (Fig. 4B).

Despite these findings, the mechanisms underlying TFRC degradation in cells remain poorly understood. Previous studies have suggested a link between TFRC ubiquitination and its iron-induced lysosomal degradation [42,43]. To explore this further, we treated cells with proteasome and lysosome inhibitors. MG132, a proteasome inhibitor, did not affect TFRC degradation, while CQ, a lysosome inhibitor, significantly prevented TFRC reduction. Notably, ART enhanced the inhibitory effect of CQ on TFRC degradation (Fig. 4C). We also examined the subcellular distribution of endogenous TFRC and lysosomes within the cells. Co-localization analysis revealed a partial overlap between TFRC and the lysosomes, further supporting the idea that TFRC undergoes lysosomal degradation (Fig. S4A). To gain a deeper understanding of how ART affects TFRC stability, we investigated its impact on TFRC distribution across subcellular compartments. ART treatment led to a marked alteration in TFRC localization, reducing its presence in late endosomes marked by RAB7 and lysosomes marked by LAMP1 (Fig. 4D and E), while increasing its accumulation in recycling endosomes marked by RAB11 and plasma membrane marked by ATP1A1 (Fig. 4F and G). To further clarify the effects of artesunate on TFRC content in different subcellular compartments, we performed subcellular fractionation experiments. This analysis revealed a significant decrease in TFRC levels in the lysosomal fraction following ART treatment, while TFRC accumulation was notably increased in both the plasma membrane and endosomal fractions (Fig. 4H). These findings suggest that artesunate treatment disrupts TFRC trafficking and alters its distribution across cellular compartments.

Further supporting these findings, we conducted molecular docking using AutoDock, which identified nine binding models with varying affinities. The best model showed a binding affinity of −7.2 kcal/mol, indicating a strong interaction between ART and TFRC. PyMOL visualization revealed three hydrogen bonds in the top model, confirming the stability of the predicted binding conformation (Fig. 4I and Fig. S4B). In line with the docking results, surface plasmon resonance (SPR) analysis demonstrated that ART interacts directly with TFRC in a concentration-dependent manner (0.3125–10 μM), further supporting the direct binding relationship between ART and TFRC (Fig. 4J).

Overall, these results provide strong evidence that ART directly binds to TFRC, preventing its lysosomal degradation and thereby modulating iron homeostasis to promote ferroptosis in gastric cancer cells.

### 3.5 HSPA9 participated in artesunate-induced cell ferroptosis

**Fig. 5:**
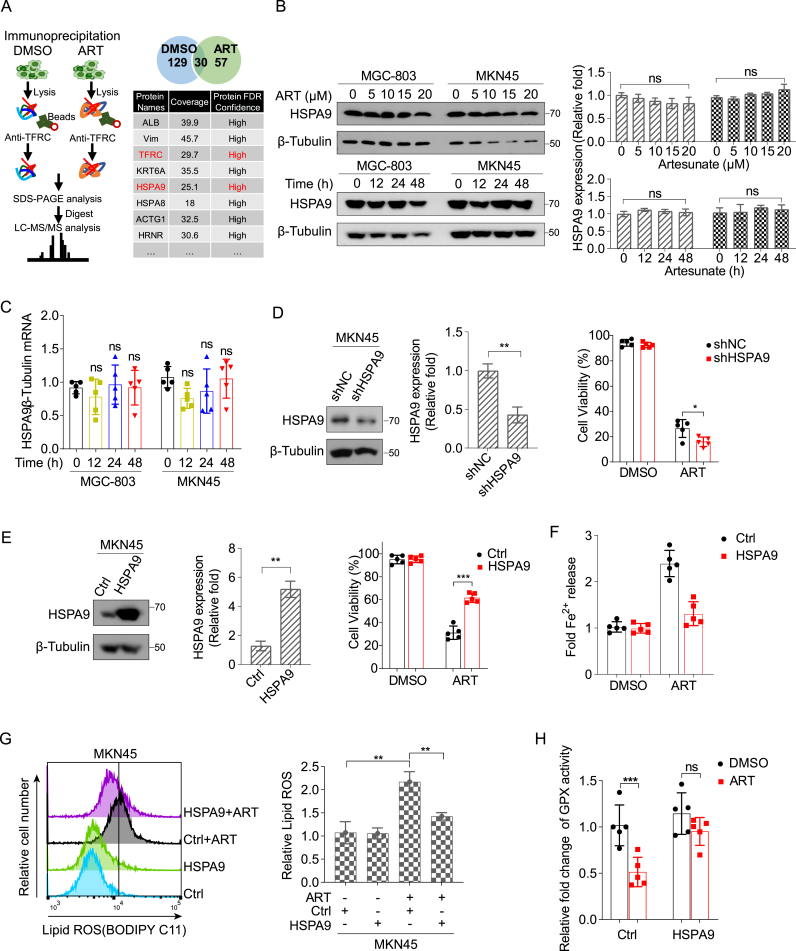
HSPA9 plays a role in the induction of ferroptosis by artesunate in cells (A) MKN45 cells treated with 15 μM artesunate for 48 h were collected using cell scrapers under cold conditions, lysed, and centrifuged at 4 °C to obtain the supernatant. TFRC and interacting proteins were immunoprecipitated using anti-TFRC antibody, followed by protein electrophoresis, concentration, and mass spectrometry analysis to identify the top 8 proteins interacting with TFRC. Data are presented as the means ± SD, n = 3 independent experiments. (B) Expression of HSPA9 in cells treated with 0–20 μM artesunate for 48 h or with 15 μM artesunate for 0–48 h, analyzed by Western blot. Data are presented as the means ± SD, n = 3 independent experiments. (C) Intracellular mRNA levels of HSPA9 in cells treated with 15 μM artesunate for 0–48 h, measured by real-time quantitative PCR. Data are presented as the means ± SD, n = 5 independent experiments. (D) Construction of a cell line with stable knockdown of HSPA9; cell viability post 72-h treatment with 15 μM artesunate. Data are presented as the means ± SD, n = 5 independent experiments. (E) The cells stably transfected with HSPA9 were treated with DMSO or 15 μM artesunate for 72 h and subjected to viability assays. Data are presented as the means ± SD, n = 5 independent experiments. (F) Ferrous ion levels in the supernatant of MKN45 cells overexpressing HSPA9, treated with 15 μM artesunate for 24 h. Data are presented as the means ± SD, n = 5 independent experiments. (G) Accumulation of lipid reactive oxygen species in MKN45 cells overexpressing HSPA9 treated with 15 μM artesunate for 24 h, evaluated by flow cytometry. Data are presented as the means ± SD, n = 3 independent experiments. (H) Glutathione peroxidase activity in MKN45 cells overexpressing HSPA9 treated with 15 μM artesunate for 48 h, measured using a kit. Data are presented as the means ± SD, n = 5 independent experiments. Significance was determined using one-way ANOVA or two-way ANOVA (*∗P* < 0.05, ∗*∗P* < 0.01, ∗∗*∗P* < 0.001).

To identify potential molecular regulators interacting with TFRC that may influence its stability and contribute to iron homeostasis and ferroptosis, we treated gastric cancer cells with either DMSO or artesunate, followed by immunoprecipitation using an anti-TFRC antibody and mass spectrometry analysis. A total of 123 proteins were identified as potential TFRC interactors. For quantitative analysis, we focused on proteins that were detected in both DMSO and artesunate-treated groups. The selection of candidate proteins was based on key mass spectrometry metrics, including low protein FDR, high confidence scores and high sequence coverage. Furthermore, we considered the biological relevance and functional characteristics of the proteins. Among the identified candidates, HSPA9 (also known as Mortalin or GRP75), a 70-kDa heat shock protein integral to cellular homeostasis, metabolic regulation, and oxidative stress response [44], emerged as a prominent candidate (Fig. 5A). Given that HSPA9 might be ruled out if it does not participate in artesunate-regulated ferroptosis, we first examined its involvement in this cell death pathway. Despite observing no changes in HSPA9 expression at the mRNA or protein levels (Fig. 5B and C), we hypothesized that modulating its expression could still impact cell sensitivity to artesunate. Stable knockdown and overexpression experiments revealed that decreasing HSPA9 significantly enhanced artesunate's cytotoxicity, while overexpression conferred a protective effect on cell viability (Fig. 5D and E). Specifically, HSPA9 overexpression reduced intracellular Fe^2+^ levels (Fig. 5F). Further investigation into the effects of HSPA9 on artesunate's cytotoxicity revealed that HSPA9 overexpression decreased lipid ROS accumulation (Fig. 5G), and increased GPXs activity (Fig. 5H). These findings indicate that HSPA9 is involved in the artesunate-induced cell death process in gastric cancer cells.

### 3.6 Artesunate hindered the degradation of TFRC by obstructing its interaction with HSPA9

**Fig. 6:**
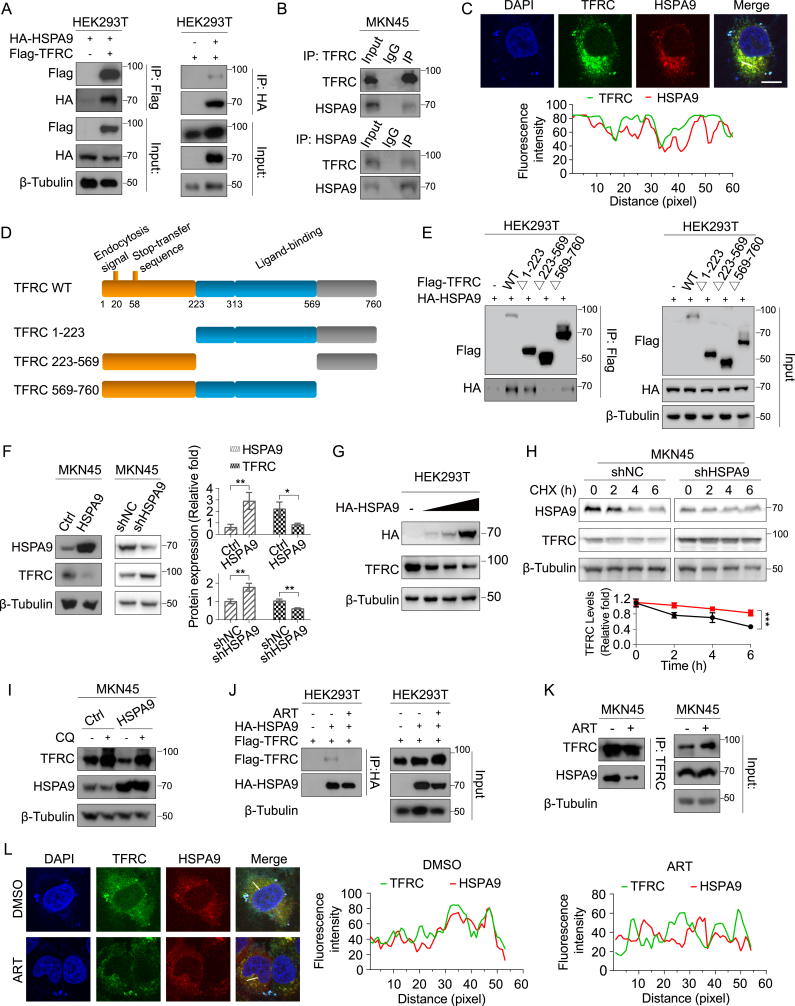
Artesunate stabilizes TFRC by Inhibiting the Interaction between HSPA9 and TFRC (A) Transfection of vectors into HEK293T cells, immunoprecipitation analysis of the interaction between Flag-TFRC and HA-HSPA9 in HEK293T cells. Data are presented as the means ± SD, n = 3 independent experiments. (B) Co-IP experiments were performed using either an TFRC antibody to pull down HSPA9 or a HSPA9 antibody to pull down TFRC in MKN45 cells. Data are presented as the means ± SD, n = 3 independent experiments. (C) Construction of mCherry-tagged HSPA9 vector (mCherry-HSPA9), transfection into MKN45 cells, DAPI staining for nuclei, and fluorescence microscopy observation of TFRC and HSPA9 colocalization. (D) Schematics for the structural domains of TFRC and the designed mutants for mapping the binding site to HSPA9. (E) Co-immunoprecipitation (Co-IP) to detect the interaction between HSPA9 and different TFRC mutants. Data are presented as the means ± SD, n = 3 independent experiments. (F) Western blot analysis of TFRC and HSPA9 expression in cell lines with stable overexpression or knockdown of HSPA9. Data are presented as the means ± SD, n = 3 independent experiments. (G) Transfection of MKN45 cells with different concentrations of HSPA9 expression vectors and subsequent Western blot analysis of TFRC expression changes after 36 h. Data are presented as the means ± SD, n = 3 independent experiments. (H) Treatment of stable HSPA9 knockdown cell lines with 2 mM CHX for 0–24 h and Western blot analysis of changes in TFRC expression. Data are presented as the means ± SD, n = 3 independent experiments. (I) Western blot detection of TFRC expression in stable HSPA9 overexpressing cell lines treated with 10 μM CQ for 48 h. Data are presented as the means ± SD, n = 3 independent experiments. (J) Transient transfection of Flag-TFRC and/or HA-HSPA9 into HEK293T cells, treatment with 15 μM artesunate for 12 h after 24 h, and Co-IP to detect changes in TFRC levels. Data are presented as the means ± SD, n = 3 independent experiments. (K) Treatment of MKN45 with 15 μM artesunate for 48 h, collection of protein supernatant, immunoprecipitation with anti-TFRC antibody to pull down TFRC protein, and Co-IP to detect changes in HSPA9 expression. Data are presented as the means ± SD, n = 3 independent experiments. (L) Transfection of mCherry-HSPA9 into MKN45 cells, treatment with 15 μM artesunate for 24 h after 12 h, DAPI staining, and fluorescence microscopy observation of TFRC and HSPA9 colocalization. Significance was determined using one-way ANOVA or two-way ANOVA (*∗P* < 0.05, ∗*∗P* < 0.01, ∗∗*∗P* < 0.001).

Next, we investigated the interaction between TFRC and HSPA9. Using HEK293T cells co-expressing Flag-tagged TFRC and HA-tagged HSPA9, coimmunoprecipitation (Co-IP) assays confirmed the interaction between these proteins (Fig. 6A). This interaction was further validated in endogenous settings, with Co-IP assays in MKN45 cells also showing TFRC-HSPA9 binding (Fig. 6B). Additionally, confocal microscopy confirmed the colocalization of TFRC and HSPA9 within these cells, supporting a functional association (Fig. 6C). To investigate the specific interaction domains between TFRC and HSPA9, we generated TFRC truncation mutants and depicted the protease-associated (PA) domain, spanning amino acids 223–569, to illustrate the segments used in our assays. Our results indicated that this domain is responsible for mediating the interaction with HSPA9 (Fig. 6D and E).

With this interaction characterized, we then explored the regulatory relationship between TFRC and HSPA9 by modulating HSPA9 expression in gastric cancer cells. Western blot analysis demonstrated that HSPA9 overexpression reduced TFRC levels, while HSPA9 knockdown led to an increase in TFRC protein (Fig. 6F). In contrast, altering TFRC expression did not impact HSPA9 levels, suggesting a unidirectional regulatory relationship (Fig. S5A). Moreover, gradient transfections with increasing concentrations of HSPA9 plasmid resulted in a dose-dependent decrease in TFRC expression, further supporting HSPA9's role in promoting TFRC degradation (Fig. 6G). To examine the impact of HSPA9 knockdown on TFRC stability, we conducted a CHX chase assay, which revealed that HSPA9 depletion extended TFRC's half-life, indicating reduced degradation (Fig. 6H). This effect was significantly mitigated by CQ, suggesting that HSPA9 primarily facilitates TFRC degradation via the lysosomal pathway (Fig. 6I).

Finally, we evaluated the effect of artesunate on the TFRC-HSPA9 interaction. Co-IP assays in both HEK293T and MKN45 cells revealed that artesunate treatment disrupted the binding between TFRC and HSPA9 in both exogenous and endogenous settings (Fig. 6J and K). Confocal microscopy corroborated these findings, showing decreased colocalization of TFRC and HSPA9 following artesunate treatment (Fig. 6L). Given our previous finding that artesunate does not alter HSPA9 expression, we investigated how HSPA9 affects TFRC distribution in response to artesunate. Molecular docking simulations showed no strong binding between artesunate and HSPA9 (Fig. S5B), suggesting artesunate does not directly interact with HSPA9. Subcellular fractionation experiments revealed that artesunate treatment reduced HSPA9 expression in endosomal compartments and abolished its presence in the lysosomal fraction, while no HSPA9 was detected at the plasma membrane (Fig. S5C). These results suggest that artesunate competes with HSPA9 for binding to TFRC, preventing HSPA9 from facilitating TFRC trafficking to the lysosome and instead promoting its recycling.

### 3.7 Artesunate effectively inhibits gastric cancer growth in vivo through TFRC-dependent ferroptosis with minimal toxicity

**Fig. 7:**
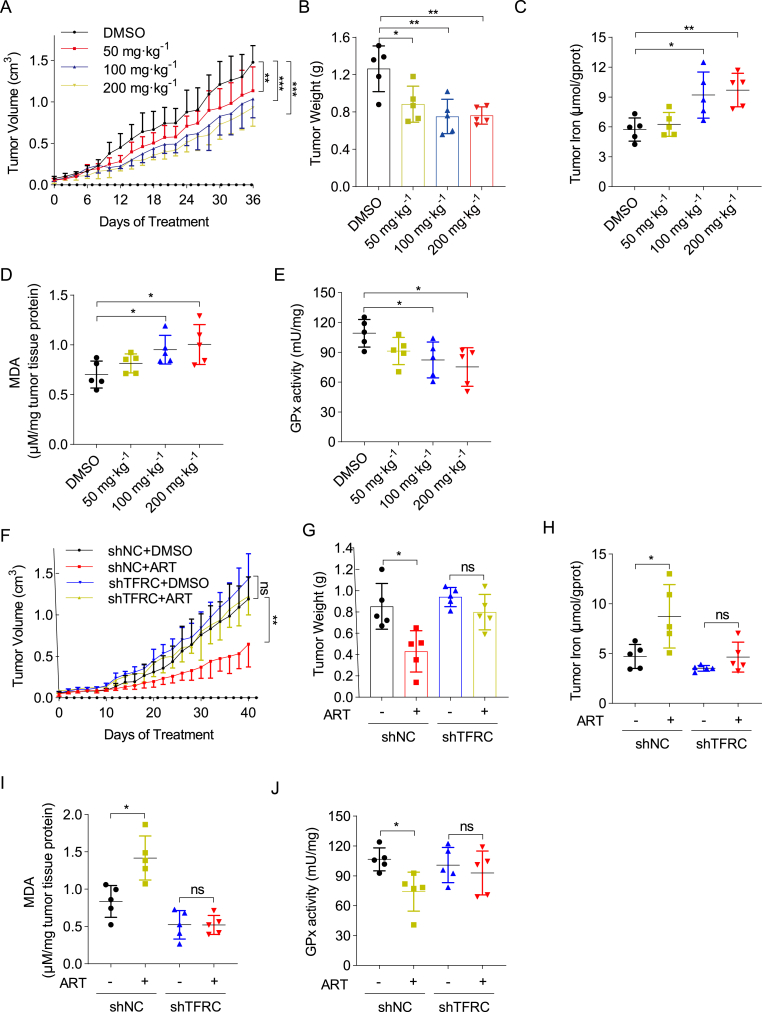
Artesunate effectively suppresses xenograft tumor growth with minimal toxic side effects and is regulated by TFRC expression (A) Intraperitoneal injection of mice with varying concentrations of artesunate every other day for 36 days. Tumor length and width measured with a caliper to calculate tumor volume and plot growth curves, n = 5 per group. (B) Post-euthanasia, extracted mouse xenografts photographed and weighed, n = 5 per group. (C) Xenografts lysed in tissue lysis buffer to measure tissue iron content, n = 5 per group. (D) Homogenized xenograft tissue lysates used for supernatant extraction and malondialdehyde level detection using an MDA assay kit. (E) GPx enzyme activity changes in xenograft tissue lysates measured using a total glutathione peroxidase assay kit. (F) Mice with shNC/shTFRC MKN45 xenografts divided into four groups, treated with DMSO or artesunate via intraperitoneal injection every other day. Tumor dimensions measured with a caliper to calculate tumor volume and plot growth curves, n = 5 per group. (G) Post-euthanasia, extracted xenografts photographed and weighed, n = 5 per group. (H) Tissue iron content measured in lysed xenografts, n = 5 per group. (I) MDA levels in xenograft tissue detected using an MDA assay kit following homogenization and supernatant extraction. (J) GPx enzyme activity changes measured in xenograft tissue lysates using a total glutathione peroxidase assay kit, n = 5 per group. Data are presented as the means ± SD; Significance was determined using one-way ANOVA or two-way ANOVA (*∗P* < 0.05, ∗*∗P* < 0.01, ∗∗*∗P* < 0.001).

To assess the therapeutic efficacy and safety of artesunate in gastric cancer treatment, we conducted an in vivo study using 20 athymic nude mice with MKN45 tumor xenografts. These mice were randomly divided into four groups and treated with vehicle control or artesunate at doses of 50 mg kg^−1^, 100 mg kg^−1^, or 200 mg kg^−1^ every other day for 36 days. Results showed that even at the lowest dose (50 mg kg^−1^), artesunate was able to inhibit tumor growth and reduced tumor weight compared to controls, highlighting its potent anti-tumor effects (Fig. 7A and B and Fig. S6A). Consistent with in vitro findings, artesunate-treated tumor tissues exhibited elevated iron content, increased malondialdehyde (MDA) levels, and decreased glutathione peroxidase (GPX) activity, indicating ferroptosis induction within the tumors (Fig. 7C–E).

To further elucidate the role of TFRC in artesunate's anti-cancer mechanism, we evaluated the effects of TFRC knockdown on artesunate-treated MKN45 cells. TFRC depletion significantly diminished the cytotoxic effects of artesunate in vivo, as evidenced by reduced tumor growth in TFRC-deficient xenografts (Fig. 7F, G and Fig. S6B). Moreover, TFRC knockdown prevented the artesunate-induced changes in iron levels, MDA production, and GPX activity, suggesting that TFRC is essential for artesunate-mediated ferroptosis (Fig. 7H–J).

Importantly, all mice tolerated artesunate treatment well, showing no significant weight loss or observable toxicity, which underscores artesunate's favorable safety profile (Fig. S6C–H). These findings indicate that artesunate is a promising therapeutic agent for gastric cancer, exerting its anti-cancer effects primarily through TFRC-dependent ferroptosis with minimal toxicity.

## 4 Discussion

Our findings highlight ferroptosis as the primary mechanism by which artesunate exerts its cytotoxic effects on gastric cancer cells, providing a targeted approach that minimally impacts normal cells. This preference for ferroptosis over apoptosis diverges from previous studies that emphasize apoptosis as the main mode of artesunate-induced cell death. By identifying TFRC as a critical mediator in this process, we offer insights into artesunate's potential for overcoming resistance in gastric cancer treatment.

While artesunate has previously been reported to induce apoptosis in various cancer types, including gastric cancer [27,45], our data indicate that ferroptosis plays a more significant role under the conditions tested. This divergence likely stems from the molecular environment within gastric cancer cells, which often feature high intracellular iron levels that can predispose these cells to ferroptosis. Iron facilitates ROS production through the Fenton reaction, leading to lipid peroxidation—a hallmark of ferroptosis. As for the observed reduction in GPX4 activity (Fig. 1H), we believe this is closely linked to artesunate-induced oxidative stress (ROS accumulation) and disruption of iron homeostasis. GPX4 is a key enzyme involved in maintaining lipid homeostasis, and its activity is highly sensitive to ROS levels. Under artesunate treatment, intracellular iron accumulation promotes the Fenton reaction, which in turn enhances ROS production. The resultant ROS accumulation may further compromise GPX4 activity, amplifying ferroptosis by impairing lipid peroxidation control. This suggests that artesunate may exert its cytotoxic effects by disrupting the delicate balance between intracellular iron levels and antioxidant defense systems, including GPX4, thereby promoting ferroptosis in gastric cancer cells.

These observations suggest that artesunate's mechanism may be better suited to environments where iron homeostasis is already skewed, as is often the case in cancer cells with heightened metabolic demands. The resulting oxidative stress surpasses the threshold for ferroptosis, while the apoptosis pathways remain only minimally engaged. This selectivity towards ferroptosis provides a distinct advantage, as it enables targeted cell death in iron-rich tumor cells, sparing normal cells and potentially reducing the side effects commonly associated with broad-spectrum chemotherapeutic agents.

Our study reveals that artesunate's cytotoxicity is critically dependent on ROS and iron homeostasis. ROS levels increased in a dose-dependent manner with artesunate treatment and could be suppressed by tiron, a superoxide scavenger and iron chelator [46]. While our pharmacological interventions with antioxidants and iron chelators provided mechanistic insights, we acknowledge that the single-dose and single-timepoint design of these experiments imposes limitations. The absence of NAC-mediated cytoprotection, while consistent with superoxide-selective effects under our experimental conditions, does not preclude potential dose-dependent modulatory effects. Future studies employing dose-response analyses and temporal profiling will be required to fully delineate the dynamic interplay between specific ROS species and artesunate's cytotoxic activity.

Furthermore, artesunate treatment increased the labile iron pool, exacerbating oxidative damage via lipid peroxidation and driving ferroptosis. The regulation of ROS and iron underscores the therapeutic potential of artesunate, as it taps into cancer cells’ vulnerability to disruptions in redox and iron balance. By amplifying these instabilities, artesunate effectively induces ferroptosis, establishing a treatment modality that aligns with the biochemical profile of cancer cells, thereby limiting collateral damage to normal tissues.

A central finding of our study is that TFRC plays a crucial role in artesunate-induced ferroptosis, primarily through enhanced iron uptake. Artesunate treatment resulted in a dose-dependent increase in TFRC protein levels, which in turn elevated intracellular iron and facilitated ferroptosis. Importantly, our molecular docking analysis indicates a potential direct interaction between artesunate and TFRC, supported by a high binding affinity and hydrogen bond formation. This interaction may stabilize TFRC on the cell surface, increasing iron availability and reinforcing the ferroptosis pathway.

Our study uncovers a novel role for the mitochondrial chaperone HSPA9 in regulating TFRC degradation through lysosomal pathways. While primarily known for its mitochondrial functions [47,48], we demonstrate that cytosolic HSPA9 binds TFRC and promotes its lysosomal degradation, establishing an unexpected extra-mitochondrial role. This interaction appears functionally critical, as HSPA9 knockdown stabilizes TFRC while overexpression accelerates its degradation - effects blocked by lysosomal inhibition. Importantly, HSPA9-mediated TFRC regulation impacts cellular iron homeostasis, where HSPA9 overexpression inhibits ferroptosis by reducing TFRC-mediated iron uptake, while its knockdown enhances artesunate-induced ferroptosis. Our Autodock molecular docking analysis shows that artesunate has a strong binding affinity for TFRC but does not interact with HSPA9. This suggests that artesunate does not directly affect HSPA9, but rather competes with HSPA9 for binding to TFRC. When artesunate binds to TFRC, HSPA9 is unable to interact with TFRC and therefore cannot escort it to the lysosome for degradation. As a result, TFRC follows its intrinsic pathway, entering recycling endosomes and returning to the plasma membrane for another round of endocytosis. Thus, while TFRC levels change due to this altered trafficking, HSPA9 remains in the cytoplasm and does not participate in the process of TFRC entering the recycling endosomes. These findings reveal HSPA9 as a key regulator of iron metabolism through post-translational control of TFRC turnover, while providing mechanistic insights into artesunate's anti-cancer effects via ferroptosis induction.

In vivo experiments reinforced our in vitro findings, with artesunate showing substantial anti-tumor activity in a TFRC-dependent manner. Artesunate-treated xenografts exhibited increased markers of ferroptosis, including elevated iron and lipid peroxidation, with minimal systemic toxicity. This selective induction of ferroptosis opens avenues for combining artesunate with other therapies that modulate iron metabolism or ROS to enhance efficacy further. The role of TFRC as a biomarker of artesunate sensitivity also suggests potential for precision medicine approaches, where patient stratification based on TFRC expression could optimize treatment outcomes.

## 5 Conclusion

This study provides a comprehensive model of how artesunate leverages TFRC stabilization and HSPA9 inhibition to induce ferroptosis in gastric cancer cells. By enhancing iron influx and ROS accumulation, artesunate selectively triggers ferroptosis, sparing normal cells and minimizing toxicity. Our findings not only expand the understanding of artesunate's mechanisms but also underscore the potential of ferroptosis-targeted therapies in gastric cancer. Future research should focus on optimizing artesunate's clinical applications, exploring synergistic combinations, and refining patient selection criteria based on TFRC and HSPA9 expression to maximize therapeutic impact.

## CRediT authorship contribution statement

**Yi Liu:** Writing – review & editing, Writing – original draft, Validation, Software, Resources, Methodology, Investigation, Formal analysis, Data curation, Conceptualization. **You Yu:** Visualization, Methodology, Formal analysis, Data curation, Conceptualization. **Zhihong Luo:** Formal analysis, Data curation, Conceptualization. **Ruoxin Fang:** Resources, Methodology, Formal analysis, Data curation. **Xiaodong Zhang:** Writing – review & editing, Writing – original draft, Software, Investigation, Data curation, Conceptualization. **Zhengkai Liao:** Visualization, Project administration, Methodology, Funding acquisition, Conceptualization. **Wenhua Li:** Writing – review & editing, Writing – original draft, Visualization, Validation, Funding acquisition, Formal analysis, Data curation, Conceptualization.

## Declaration of competing interest

The authors declare the following financial interests/personal relationships which may be considered as potential competing interests: Wenhua Li reports financial support was provided by the 10.13039/501100001809National Natural Science Foundation of China. Wenhua Li reports financial support was provided by 10.13039/501100004791Shenzhen Science and Technology Program. Wenhua Li reports financial support was provided by the Translational Medicine and Interdisciplinary Research Joint Fund of 10.13039/501100016359Zhongnan Hospital of Wuhan University. If there are other authors, they declare that they have no known competing financial interests or personal relationships that could have appeared to influence the work reported in this paper.

## Data Availability

Data will be made available on request.
