# Supplementary material for: Artesunate induces ferroptosis in gastric cancer by targeting the TFRC-HSPA9 axis for iron homeostasis regulation
Source: Redox Biol. 2025 Sep 10;87:103867. doi: 10.1016/j.redox.2025.103867 (PMC12628023; doi:10.1016/j.redox.2025.103867)
Supplement: Multimedia component 3 [file mmc3.pdf]

Supplementary Figure 1

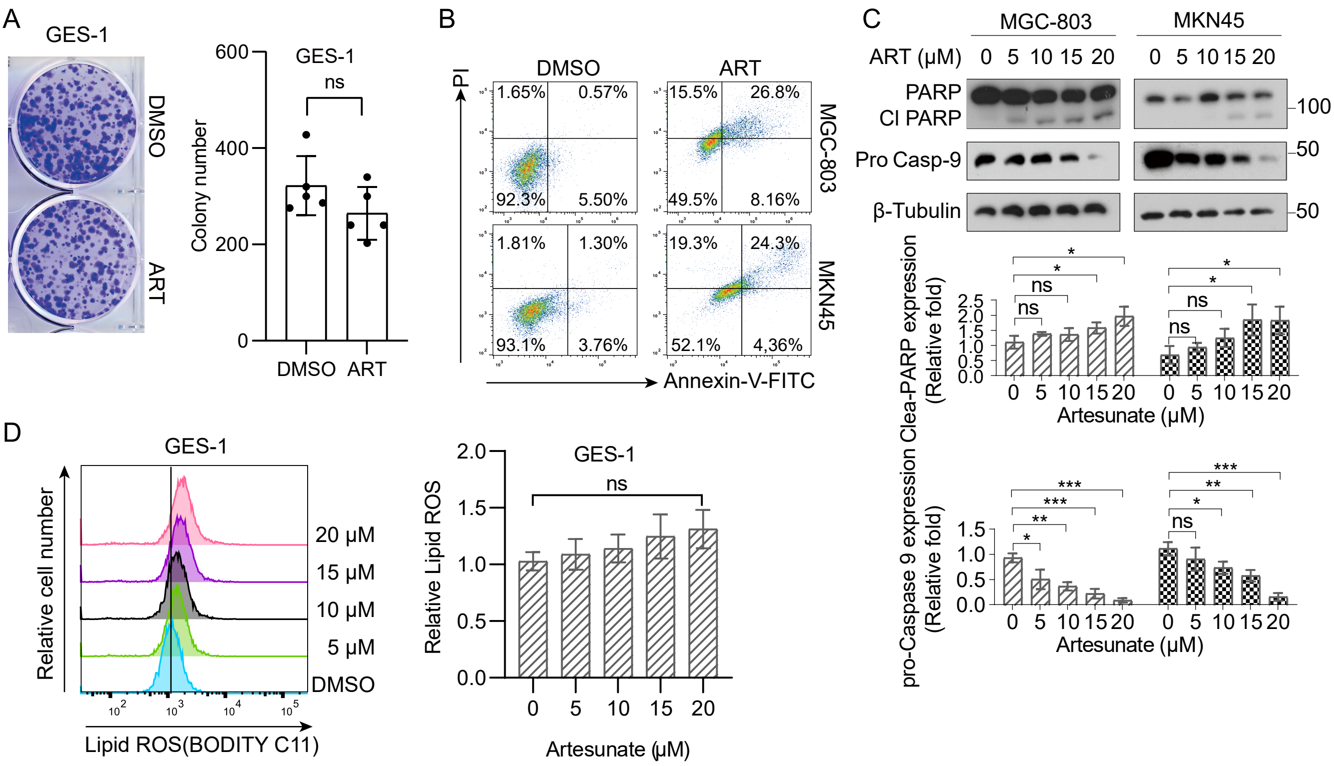

Figure S1 Artesunate Predominantly Induces Ferroptosis in Gastric Cancer Cells

(A) Colony formation assay of GES-1 cells treated with artesunate for 6 d. After staining with crystal violet, the number of colonies was quantified.

(B) Apoptosis was measured by flow cytometry after 48 h of 20 μM artesunate treatment.

(C) Western blot analysis was performed to assess the cleavage status of the apoptotic-related proteins PARP and Caspase 9 in both cell lines following the same treatment.

(D) GES-1 cell lines treated with varying concentrations of artesunate for 24 hours undergo flow cytometry to detect changes in intracellular lipid ROS.

Data are presented as the means ± SD, n = 3 independent experiments; Significance was determined using one-way ANOVA or two-way ANOVA (*$P < 0.05$, **$P < 0.01$, ***$P < 0.001$).

Supplementary Figure 2

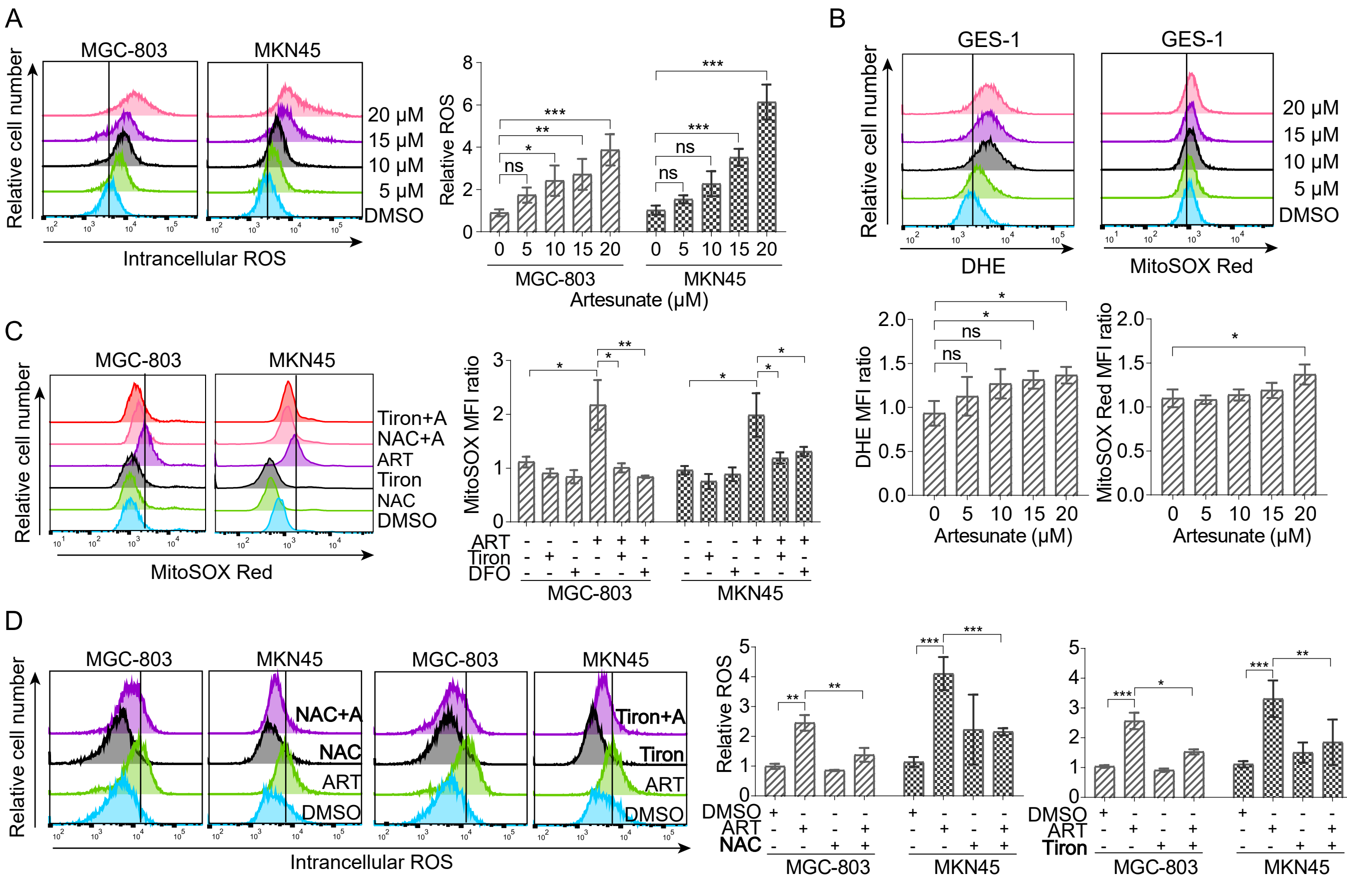

Figure S2 Artesunate-induced Oxidative Stress and Iron Homeostasis Disruption Promote Ferroptosis in Gastric Cancer Cells.

(A) Accumulation of total ROS clusters (detected by DCFH-DA) in MGC-803 and MKN45 cells treated with various concentrations of artesunate for 24 hours, assessed via flow cytometry.

(B) The levels of intracellular superoxide (detected by DHE) and mitochondrial superoxide (detected by MitoSOX Red) were assessed by flow cytometry. Representative flow cytometric histograms (left panel) and quantitative analysis of the MFI ratio (right panel) are shown.

(C) The levels of mitochondrial superoxide (detected by MitoSOX Red) were assessed by flow cytometry.

(D) ROS accumulation in cells pre-treated with 10 mM NAC or 1 mM Tiron for 3 hours, followed by 24-hour treatment with 15 μM artesunate, measured using flow cytometry.

Data are presented as the means ± SD, n = 3 independent experiments; Significance was determined using one-way ANOVA or two-way ANOVA (*P < 0.05, **P < 0.01, ***P < 0.001).

Supplementary Figure 3

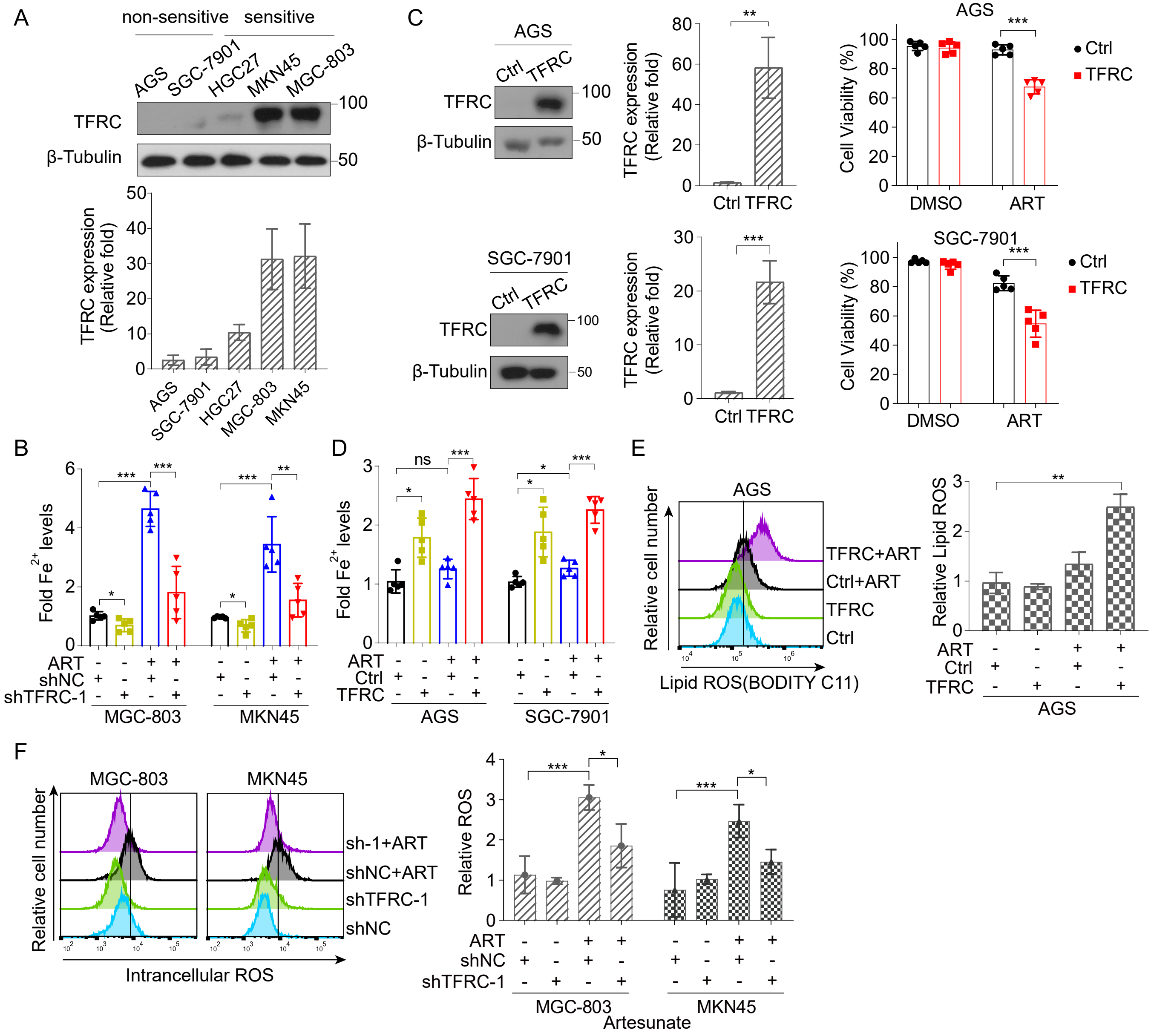

Figure S3 The Role of TFRC in Artesunate-Induced Cell Death in Gastric Cancer Cells.

(A) Collection of various gastric cancer cells, protein extraction followed by western blot analysis of TFRC expression.

(B) Effects of ART (artesunate) on Fe^{2} levels in MGC-803 and MKN45 cells. Cells were transfected with shNC (control) or shTfrc-1 and then treated with ART (+) or not (-). Fe^{2} levels were measured and presented as fold change.

(C) Construction of TFRC overexpression vectors, transfected into low TFRC-expressing AGS and SGC-7901 cell lines, with overexpression efficiency verified by western blot; cell viability in the overexpressing cell lines treated with 15 μM artesunate for 72 hours, assessed using trypan blue staining.

(D) Effects of artesunate (ART) on intracellular labile Fe^{2} levels in AGS and SGC-7901 cells. Cells were treated with ART or control (Ctrl) in the presence or absence of TFRC. Fe^{2} levels were measured and presented as fold change relative to control.

(E) Changes in lipid reactive oxygen clusters in stable TFRC overexpressing AGS cell lines treated with 15 μM artesunate 24 hours, assessed by flow cytometry.

(F) Quantification of intracellular ROS levels in stable TFRC knockdown cell lines treated with 15 μM artesunate for 24 hours. Data are presented as the means ± SD, n = 3 independent experiments; Significance was determined using one-way ANOVA or two-way ANOVA (*P < 0.05, **P < 0.01, ***P < 0.001).

Supplementary Figure 4

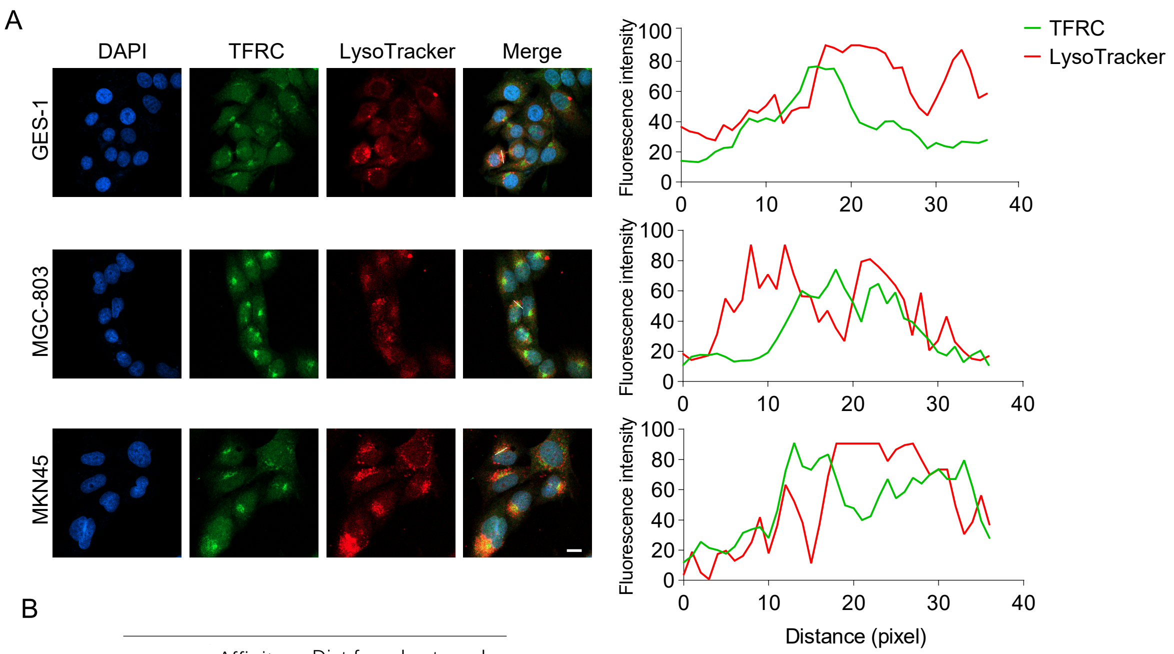

B

Mode	Affinity (kcal/mol)	Dist from best mode rmsd l.b.	rmsd u.b.
1	-7.2	0.000	0.000
2	-7	1.833	4.053
3	-6.8	1.577	3.663
4	-6.7	11.92	15.82
5	-6.6	18.148	21.691
6	-6.6	23.994	25.609
7	-6.6	2.447	4.765
8	-6.4	10.299	12.814
9	-6.4	32.694	35.706

Figure S4 Artesunate Modulates TFRC Stability and Lysosomal Degradation to Promote Ferroptosis in Gastric Cancer Cells

(A) Representative images showing TFRC (green) and lysosomes (red, stained with LysoTracker) in GES-1, MGC-803, and MKN45 cells, with nuclear staining by DAPI (blue). Merge images indicate the co-localization of TFRC and lysosomes. The right panels show fluorescence intensity profiles along a line drawn through the merged images, comparing TFRC and MitoTracker staining intensities. Scale bar = 5 μm.

(B) Nine binding modes of TFRC and artesunate were obtained using AutoDock. Mode 1 displayed the strongest binding affinity of -7.2 kcal/mol with no deviation from the best mode, while other modes showed varying affinities and rmsd values, indicating multiple potential binding conformations.

Supplementary Figure 5

A

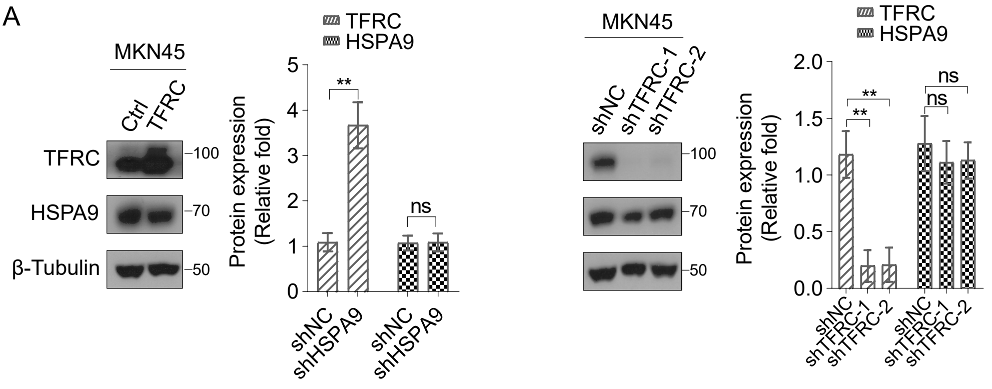

B

Mode	Affinity (kcal/mol)	Dist from best mode rmsd l.b.	Dist from best mode rmsd u.b.
1	-0.0	0.000	0.000
2	-0.0	33.959	36.218
3	-0.0	28.986	31.968
4	-0.0	35.621	37.313
5	-0.0	22.605	25.486
6	-0.0	34.145	35.905
7	-0.0	35.826	38.680
8	-0.0	20.628	24.487
9	-0.0	12.289	15.569

C

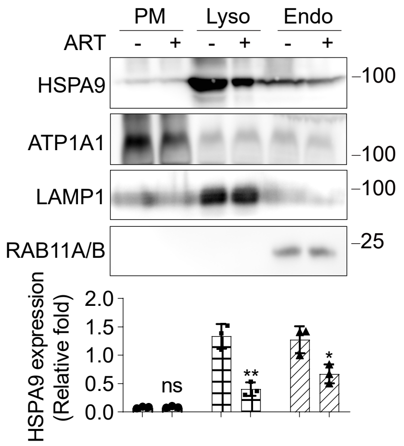

Figure S5 Artesunate stabilizes TFRC by disrupting the interaction between HSPA9 and TFRC

(A) Western blot analysis of HSPA9 expression in cell lines with stable overexpression or knockdown of TFRC.

(B) The table summarizes the docking results for nine binding modes between HSPA9 and artesunate. Mode 1 shows the highest binding affinity of -0.0 kcal/mol, with no deviation from the best mode (rmsd l.b. 0.000, rmsd u.b. 0.000).

(C) Cells were fractionated using the Subcellular Protein Fractionation Kit (BestBio, China) according to the manufacturer's instructions. Lysosomal, endosomal, and plasma membrane fractions were isolated through sequential centrifugation steps. The resulting protein fractions were collected, quantified, and stored at -80℃ for subsequent Western blot analysis.

Data are presented as the means ± SD, n = 3 independent experiments; Significance was determined using one-way ANOVA or two-way ANOVA (**P* < 0.05, ***P* < 0.01, ****P* < 0.001).

Supplementary Figure 6

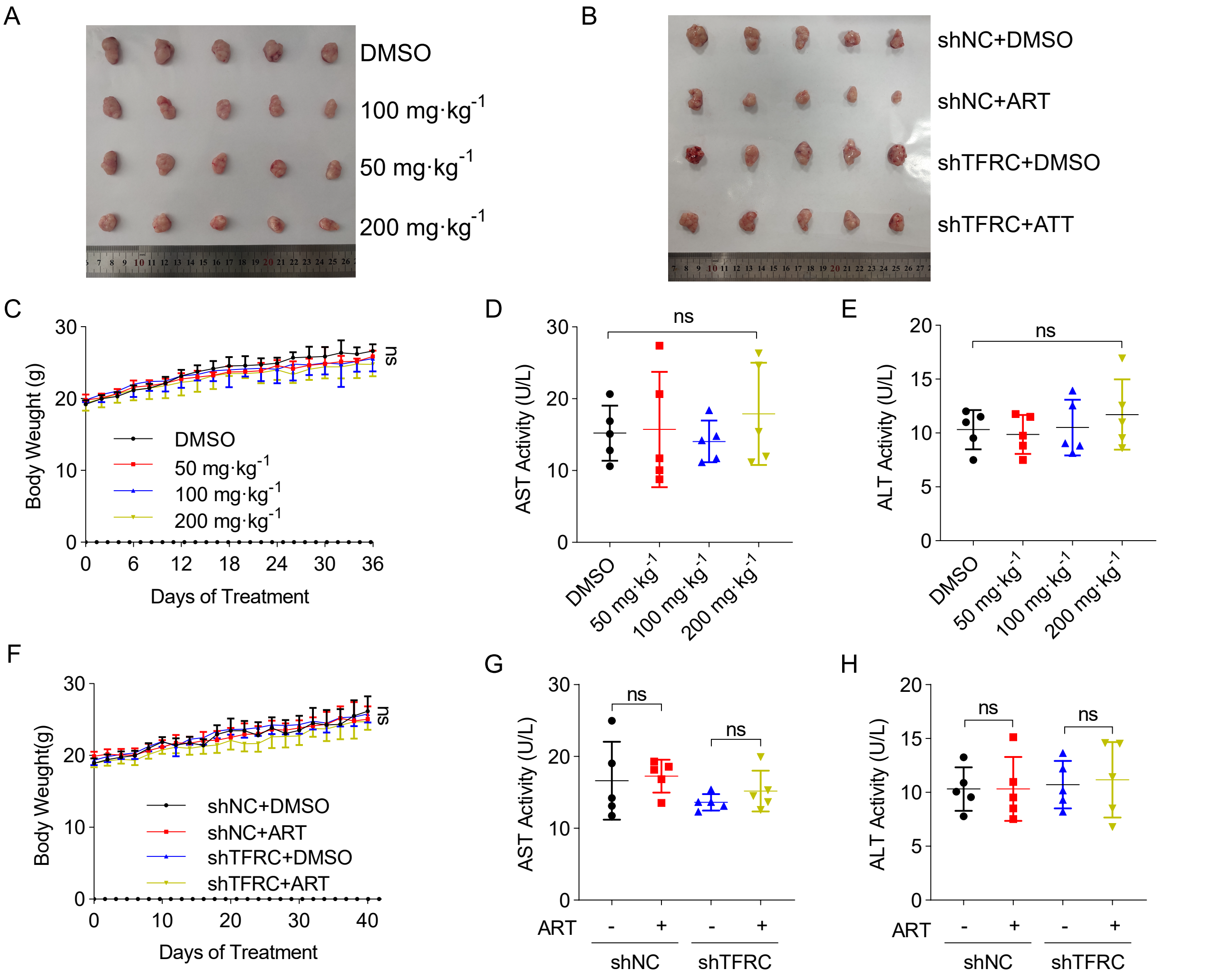

Figure S6. Artesunate suppresses tumor growth with minimal toxicity, regulated by TFRC expression.

(A) Post-euthanasia, xenograft tumors extracted from mice treated with DMSO, 50 mg/kg, 100 mg/kg, and 200 mg/kg artemether, photographed and weighed, n = 5 per group.

(B) Post-euthanasia, xenograft tumors extracted from mice in shNC+DMSO, shTFRC+DMSO, shNC+ART, and shTFRC+ART groups, photographed and weighed, n = 5 per group.

(C) Intraperitoneal injection of artesunate in four groups of mice, administered bi-daily, with concurrent monitoring and plotting of mouse body weights, n = 5 per group.

(D) Blood collection from mice prior to euthanasia for assessment of serum aspartate aminotransferase (AST) activity, n = 5 per group.

(E) Blood collection from mice prior to euthanasia for assessment of serum alanine aminotransferase (ALT) activity, n = 5 per group.

(F) Regular monitoring and recording of body weights of mice in the four groups mentioned in (E), with graphical representation.

(G-H) Assessment of serum AST and ALT activity in the four groups of mice mentioned in(B).

Data are presented as the means ± SD; Significance was determined using one-way ANOVA or twoway ANOVA (**P* < 0.05, ***P* < 0.01, ****P* < 0.001).
